# Global status of 47 major wheat loci controlling yield, quality, adaptation and stress resistance selected over the last century

**DOI:** 10.1186/s12870-018-1612-y

**Published:** 2019-01-03

**Authors:** Junjie Zhao, Zhiwei Wang, Hongxia Liu, Jing Zhao, Tian Li, Jian Hou, Xueyong Zhang, Chenyang Hao

**Affiliations:** Key Laboratory of Crop Gene Resources and Germplasm Enhancement, Ministry of Agriculture/The National Key Facility for Crop Gene Resources and Genetic Improvement/Institute of Crop Sciences, Chinese Academy of Agricultural Sciences, Beijing, 100081 China

**Keywords:** Bread wheat, Agronomic traits, Selection, KASP marker, Breeding

## Abstract

**Background:**

Wheat breeding over the last 100 years has increased productivity by adapting genotypes to local conditions, but the genomic changes and selection signals that caused phenotypic change during breeding are essentially unknown. Studying and understanding human selection of multiple important genes controlling key phenotypic traits will promote wheat molecular breeding.

**Results:**

A total of 1152 diverse global wheat materials were genotyped based on KASP markers from 47 genes controlling grain yield, grain quality, adaptation, and stress resistance. Significant phenotypic variations between landraces and modern cultivars were found in 11 adaptive and yield-related traits. Thirty-six improvement-selective favorable alleles, including 22 positive prolonged and 14 negative selection alleles, were identified through comparing frequency spectra. *Sus1-7A-Hap-H*, *Sus1-7B-Hap-T*, *Sus2-2A-Hap-A*, *TGW6-A1a*, *Cwi-4A-Hap-C*, *vrn-A1*, *PHS1-PHS+* and *Lr34+* were subjected to strong selection, and overwhelmingly strong selection had occurred before improvement selection at *Psy-A1b*, *Psy-B1a or b*, *Psy-D1a* and *Cwi-5D-Hap-C*. However, *Rht-B1b*, *Rht-D1b* and *1BL.1RS* were rare or absent in Chinese landraces but present in modern Chinese cultivars and introduced accessions. Importantly, *Lr68+*, *Fhb1+*, *Wx-B1b* and *Yr15+*, currently existing at a low frequency, should be regarded as further major improvement targets in global wheat breeding. Gene flow analysis showed that introduced cultivars especially from the former USSR and Italy contributed to enriched genetic variation in modern Chinese cultivars.

**Conclusions:**

This work objectively reports human selection on favorable alleles of multiple crucial genes in Asia, Europe, North America and CIMMYT, and traces the distribution of important genes in global wheat for molecular breeding.

**Electronic supplementary material:**

The online version of this article (10.1186/s12870-018-1612-y) contains supplementary material, which is available to authorized users.

## Background

Bread wheat (*Triticum aestivum* L.), one of the most important crops worldwide for human nutrition, originated 8000–10,000 years ago [1, 2]. With the rapid development of agriculture over the last 100 years, breeders have increased grain yield by adapting genotypes to local conditions [3] and have utilized variation in many traits [4]. Improved traits cover a wide range, such as semi-dwarf statures [5], vernalization and photoperiod responses [6], tolerance to biotic and abiotic stresses [7], nutritional quality, and yield stability [8].

Chinese wheat production has made significant advances since 1949. This began with utilization of superior landraces in the early 1950s and direct or indirect exploitation of introduced varieties. Landraces formed the basis for wheat improvement programs [9] and were valuable sources of genetic diversity and adaptation to local environmental conditions [10]. Landraces were key genetic resources for modern wheat breeding and provided the basic genetic materials for variety improvement [4, 11]. Introduced varieties also played major roles in improving wheat production and genetic diversity. Introduced cultivars contained 76.3% of the alleles present in Chinese accessions, and some of these alleles are correlated with known functional genes controlling traits such as resistance to leaf rust and stripe rust, and high grain yield [12]. Therefore, analysis of genetic variation in wheat on a global scale could allow breeders to identify further desirable germplasm for breeding.

The detection of past selected loci, as one method of evaluating genetic change might identify breeding targets and provide opportunities for improving genomic selection models [13, 14]. Genomic studies have identified loci during improvement in soybean [15], cotton [16], rice [17, 18] and maize [19]. With the application of single nucleotide polymorphism (SNP) chips, some selected regions and haplotype blocks associated with important agronomic traits were identified in wheat [3, 20–23]. These efforts have an impressive potential impact to help us understand improvement-selective sweeps in bread wheat, but with relatively higher cost and false-positive rate. Crop improvement involves artificial selection of specific alleles at loci controlling key morphological and phenotypic traits [4]. Therefore, varieties experienced strong selection at genes identified as targets of artificial selection [24]. Moreover, functional genes have not been used to uncover genetic variations and selection signals during wheat improvement in a sufficient number of samples. Fortunately, more than 100 functional markers from cloned genes are available for agronomic traits in wheat [25], and with the advance of high-throughput genotyping, many kompetitive allele specific PCR (KASP) assays for those genes have been developed and validated [26]. The KASP genotyping technology of LGC Genomics [26, 27] is a flexible genotyping platform for discrimination of SNP or InDel differences. Therefore, an excellent opportunity has been created for assessing the genome-wide effects of functional genes selected during wheat breeding.

In this study, a large, diverse collection of 1152 wheat accessions representing a worldwide geographic distribution were analyzed using KASP assays from 47 genes controlling agronomic traits including grain yield, quality, adaptation, and stress resistance. Genetic diversity and divergence were estimated on both phenotypic and genotypic differences between landraces and modern cultivars in order to detect selection signals during improvement. In addition, gene flow and allelic variation in functional genes were compared to demonstrate the contributions of introduced accessions to Chinese wheat breeding. This study provides a robust foundation for identifying targets of selection and understanding global distribution of important genes for wheat molecular breeding.

## Methods

### Plant materials and DNA extraction

To ensure wide geographical distribution and a rich representative sampling of wheat germplasm we selected a total of 1152 accessions including 672 introduced cultivars and 480 Chinese varieties (Additional file 1: Table S1). The latter set included 245 accessions in the mini-core collection [28] and 235 modern cultivars released over the three decades from 1980 to 2010. The 672 introduced accessions originated from four international regions covering 21 countries, including North America (153), CIMMYT (53), Europe (384) and the former USSR (82). Genomic DNA was isolated from fresh leaves of a single plant in each accession by the CTAB method [29]. The final diluted DNA concentration was 40 ng/μL.

### Phenotypic assessment and statistical analyses

During the wheat-growing seasons from 2014 to 2016, the 480 Chinese accessions were planted at the Chinese Academy of Agricultural Sciences (CAAS) Xinxiang Experiment Station in Henan province (113.5°E, 35.2°N). The planting density was 40 seeds per row in 2 m four-row plots, and the rows were 25 cm apart. Field management followed local practices. Ten plants from the middle row of each accession were measured to evaluate eleven traits, including heading date (HD, days), flowering date (FD, days), plant height (PH, cm), effective tiller number (ETN, number), spike length (SL, cm), spikelet number per spike (SN, number), kernel number per spike (KN, number), 1000-kernel weight (TKW, g), kernel length (KL, mm), kernel width (KW, mm) and kernel thickness (KT, mm).

Basic statistics and ANOVA using the Tukey test between populations for each trait at a significance level of 5% (*P* ≤ 0.05) were carried out using PROC MIXED in SAS v.9.4 (SAS Institute 2010). We used values calculated by the best linear unbiased predictor (BLUP) method [30] to represent the mean value of each trait from different years. The overall mean (X) and standard deviation (σ) were subdivided by the accession value into 10 frequency classes (X_i_) ranging from the first (X_i_<X-2σ) to the tenth (X_i_>X + 2σ) with 0.5σ spacing. The phenotypic diversity of each trait for different subpopulations was estimated using the Shannon-weaver index (H) [31].

$$ \mathrm{H}=-\sum \limits_{\mathrm{i}=1}^{\mathrm{s}}{\mathrm{P}}_{\mathrm{i}}\ln {\mathrm{P}}_i $$

Where P_i_ represents the ratio of the number of the accessions in the corresponding class to the total based on phenotypic data.

### KASP genotyping of functional genes

Liu et al. [25] summarized available functional markers based on more than 30 cloned wheat genes controlling grain yield, quality, adaptation and stress resistance. Then, Rasheed et al. [26] converted these conventional functional markers into KASP assays that were confirmed to be reliable, accurate and significantly associated with the relevant phenotypes in the cultivars and segregating populations. In this study, the robust 52 KASP assays derived from 47 cloned genes underpinning key agronomic traits were used for genotyping (Additional file 1: Table S2). Information for seven assays associated with grain yield were provided by Dr. Awais Rasheed (unpublished). Coding sequences for other KASP assays were obtained from the literature and used to design KASP primers based on diagnostic SNPs following standard KASP guidelines. The allele-specific primers carried the standard FAM (5’ GAAGGTGACCAAGTTCATGCT 3′) and HEX (5’ GAAGGTCGGAGTCAACGGATT 3′) tails on the targeted SNP at the 3′ end. A common primer was designed so that the total amplification length was less than 120 bp [26].

KASP assays were performed in 384-well formats with 5.0 μL mixtures containing 2.2 μL of 40 ng/μL DNA, 2.5 μL of 1 X KASP V4.0 2X Master mix (KBS-1016-017), 0.04 μL Mg^2+^, 0.056 μL of primer mixture, and 0.204 ddH_2_O. Ultrapure water was used as the non-template control (NTC). PCR was performed with the following profile: hot start at 95 °C for 15 min, followed by ten touchdown cycles (95 °C for 20 s; touchdown at 65 °C initially and decreasing by 1 °C per cycle for 25 s), followed by 30 additional cycles of annealing (95 °C for 10 s; 57 °C for 60 s). Amplification products were genotyped on QuantStudio™ 7 Flex (Applied Biosystems by Life Technologies), and data were visualized using QuantStudio™ Real-time PCR software v.1.3 (Applied Biosystems by Life Technologies).

### Population structure and phylogenetic analysis

Genotypic data of 47 genes was used to calculate genetic distance among the 1152 accessions using Nei’s distances [32]. A neighbor-joining tree was constructed in PowerMarker v3.25 [33]. MEGA 5 [34] was used to visualize the phylogenetic tree. The parameters for genetic diversity were conducted using PowerMarker v3.25. Principal coordinate analysis (PCA) was also performed to reveal relationships among different populations using the R package Adegenet v2.0.1 [35], and the first two eigenvectors were plotted in two populations.

### Analyses of association between KASP markers and traits

Using phenotypic data for 11 traits in three environments, we detected significant associations between loci and traits. According to population structure above, KASP assays from 47 genes of Chinese landraces and modern Chinese cultivars were respectively used to implement association analyses with one-way ANOVA using PROC MIXED in SAS v.9.4 (SAS Institute 2010), and a significant association was declared at a threshold of *P*<0.05.

### Estimation of genetic differentiation and selection signals

Divergences, *F*-statistics (*Fst*) and genetic distance, are measures of population differentiation based on genetic polymorphism data [36]. We also analyzed gene flow between subpopulations from different countries [37]. Divergence and gene flow were calculated with POPGENE software [38]. To detect improvement loci from landraces to modern cultivars, selection signals were estimated by altering the population allelic frequency of polymorphic loci of the target genes [17, 39]. A frequency difference between the two subgroups was interpreted to be an important selection criterion using Z tests in SAS software (SAS Institute 2010). This has been used to measure population differentiation in animals [40] and soybeans [41].

## Results

### KASP assays and genetic structure in global wheat resources

**Fig. 1:**
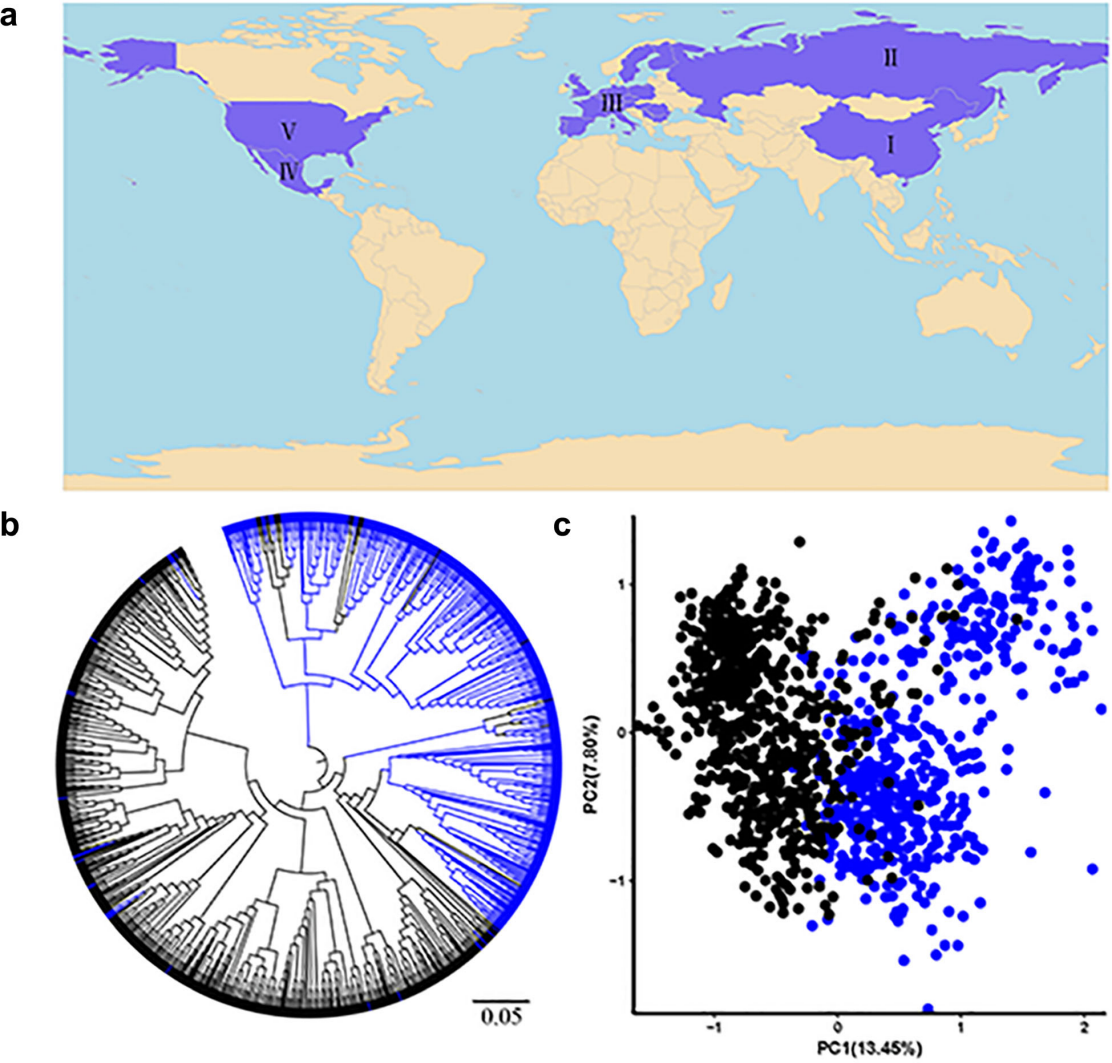
Geographic distribution and phylogenetic relationships of 1152 accessions. **a** Geographic sources in which the distributed regions are marked in purple. The five regions are China, former USSR, Europe, CIMMYT and North America presented as I–V, respectively. The world map is available at https://commons.wikimedia.org/wiki/Category:Blank_SVG_maps_of_the_world. **b** A neighbor-joining tree of 1152 accessions constructed with 47 KASP markers. **c** PCA plots of all accessions based on the same number of markers. Chinese and introduced accessions are shown in blue and black, respectively

The 1152 accessions were evaluated for genotype, phenotype and geographical distribution (Fig. 1a; Additional file 1: Table S1). Four hundred and eighty accessions were selected from the Chinese mini-core collection and recently released Chinese cultivars; 672 accessions were introduced from other countries (Additional file 2: Figure S1).

Genotyping of the entire collection using 47 functional genes allowed us to identify the allelic variations (Additional file 1: Table S1). The polymorphic assays from 47 loci were classified into four trait categories relating to grain yield, quality, adaptation, and stress resistance. Phylogenetic analysis (Fig. 1b) and principal component analysis (PCA) (Fig. 1c) showed that lines were divided into two groups referred to as Chinese wheat resources and introduced cultivars (IC), although different degrees of introgression were detected between them. Accessions from China were divided into two subgroups, Chinese landraces (CL) and modern Chinese cultivars (MCC) (Additional file 3: Figure S2). Introduced accessions were subdivided into four subgroups, viz. European, former USSR, CIMMYT and North American (Additional file 4: Figure S3).

### Phenotypic variations from landraces to modern Chinese cultivars

Morphological features were primarily selected during improvement, resulting in significant phenotypic differences between landraces and modern cultivars. The Chinese wheat panel of 157 landraces and 323 modern cultivars was evaluated for 11 grain yield and adaptive traits in three environments based on genetic structure of Chinese wheat. Basic statistics for all traits based on BLUP value indicated significant changes in each trait (*P*<0.001) (Additional file 1: Table S3). We made histograms of the 11 traits in different environments (Additional file 5: Figure S4) and found that in comparison to landraces, heading (HD) and flowering (FD) dates of modern cultivars were earlier and plant height (PH) was reduced. Kernel number per spike (KN), 1000-kernel weight (TKW), kernel length (KL), kernel width (KW) and kernel thickness (KT) were significantly increased, but effective tiller number (ETN), spike length (SL), spikelet number per spike (SN) were decreased in modern cultivars. The trends of differences at all traits between two groups were stable in three years (Additional file 6: Figure S5).

### Modern breeding promotes divergence of functional genes from landraces to modern cultivars

Due to significant phenotypic differences between landraces and modern cultivars, we estimated the genetic diversity between them on 47 genes (Additional file 7: Figure S6a). The mean diversity of the MCC estimated at 0.32 was significantly higher than that of the CL (0.26) (*P*<0.05). The diversity of functional genes increased gradually during successive decades of breeding (Additional file 7: Figure S6b). This suggested that conventional breeding by artificial hybridization has increased diversity in gene coding regions. Population genetic differentiation measured by Wright’s *Fst* and genetic distance further revealed that genetic divergence occurred during breeding (Additional file 7: Figure S6c). These results indicate that modern breeding has been increasing the divergence in functional genes between landraces and modern cultivars.

### Conjoint analysis of improvement-selective signatures and association signals

**Fig. 2:**
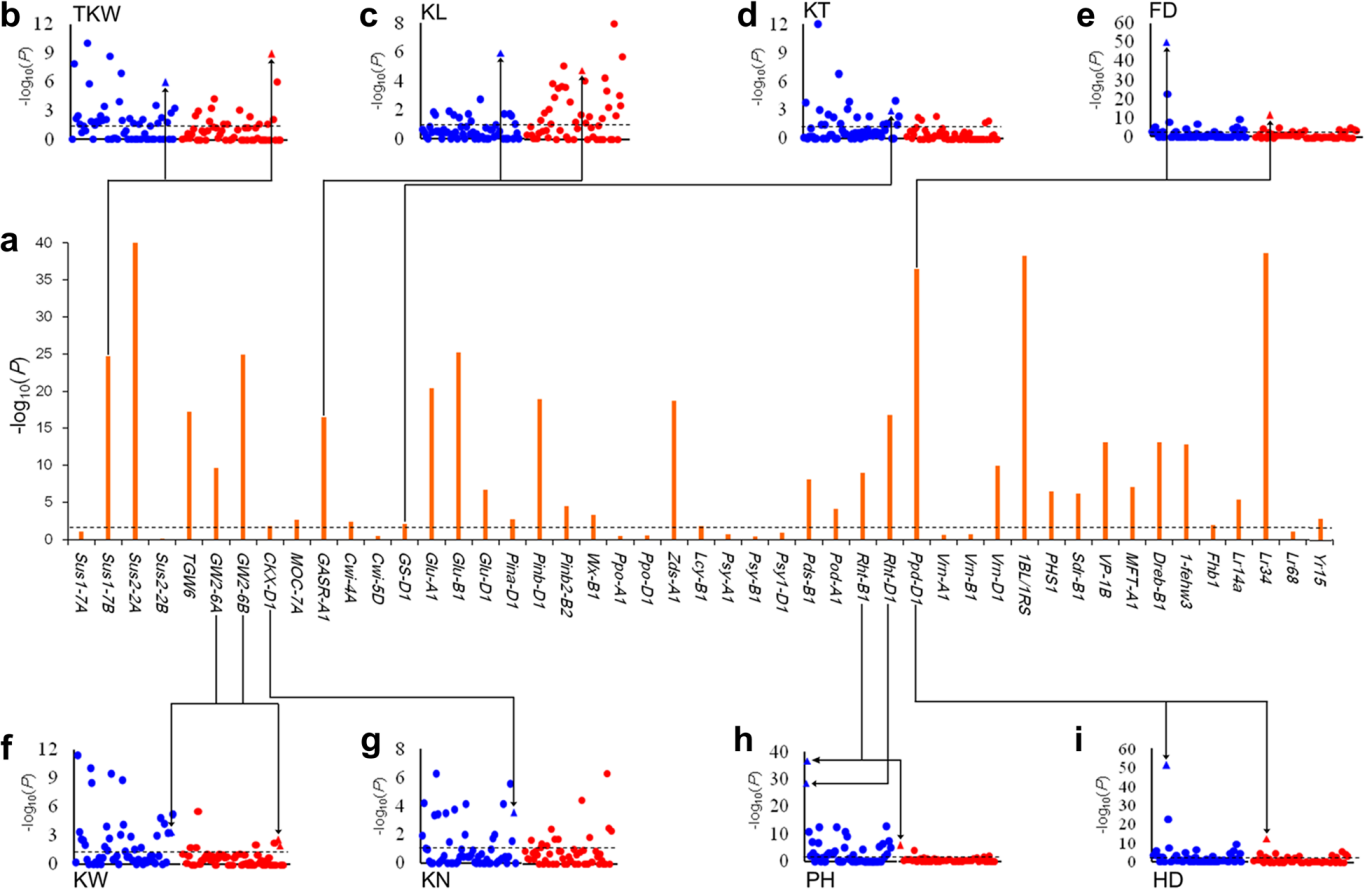
Improvement-selective sweeps detected by comparisons between Chinese landraces (CL) and modern Chinese cultivars (MCC). **a** Whole-genome screening of selection signals of wheat improvement in 47 genes controlling important agronomic traits detected by comparisons of allele frequency between CL and MCC using Z tests. Horizontal dashed lines indicate genome-wide thresholds of selection signals (Z test, −log_10_(*P*) = 1.3). **b–i** Association signals corresponding to the relevant traits overlapping with strong selective signals. The blue and red dots represent associated signals in MCC and CL, respectively. The horizontal dashed gray lines indicate significance thresholds of association signals. The arrows mean loci associated with respective target traits

**Fig. 3:**
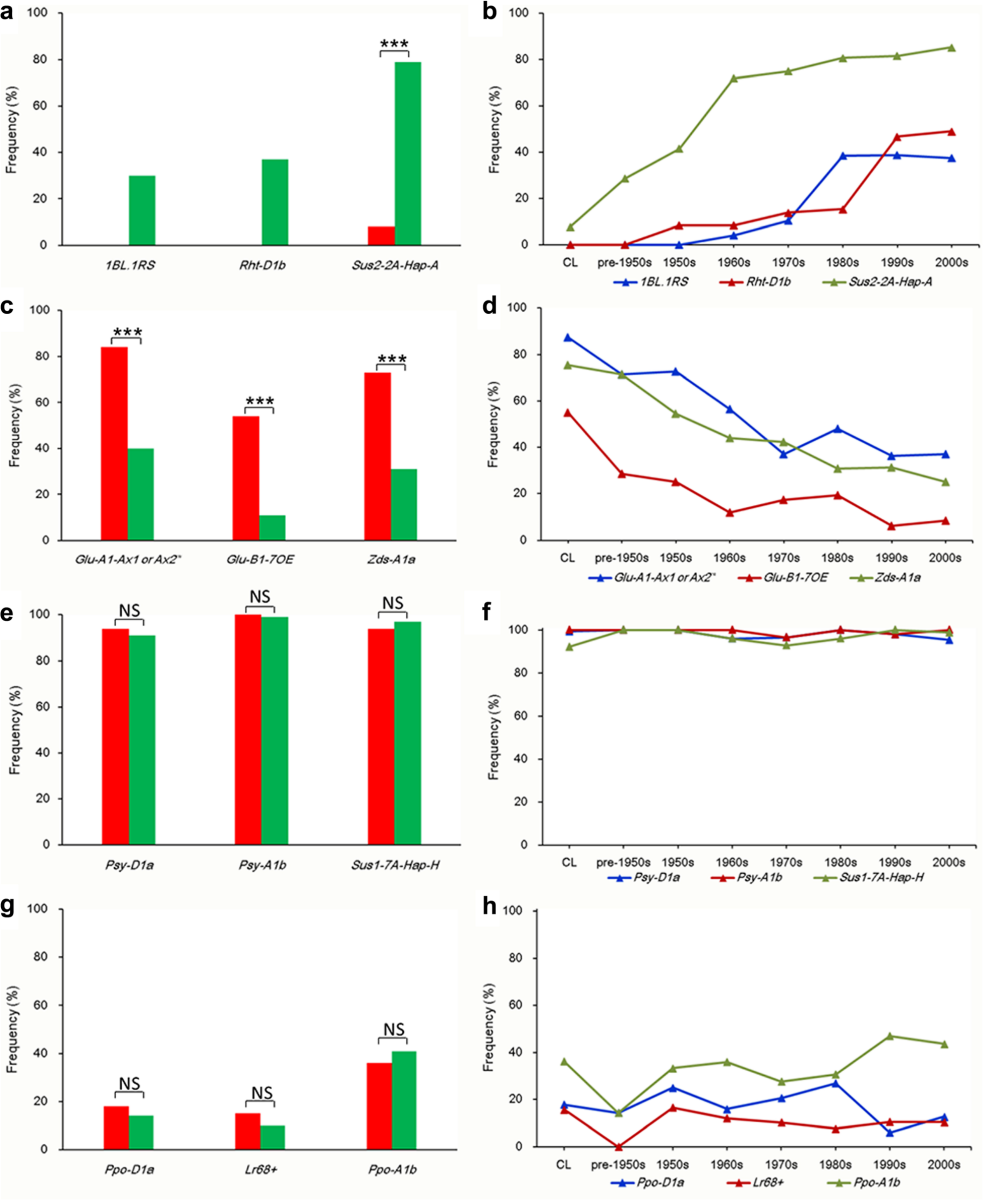
Allele frequencies of Chinese landraces (CL) and modern Chinese cultivars (MCC). Red histograms indicate CL, and green shows MCC. **a-b** Favorable allele frequencies increasing significantly from CL to MCC during different decades. **c-d** Favorable allele frequencies decreasing significantly from CL to MCC during different decades. **e-f** High favorable allele frequencies in both CL and MCC. **g-h** Low favorable allele frequencies in both CL and MCC. ***P<0.001; NS: not significant

Genetic divergence caused the frequency spectra divergence of selected loci. Thirty six of 47 functional genes had significant differences in allele frequency between CL and MCC (*Z* test, *P*<0.05) (Fig. 2a). Differences in the allele frequencies of the 36 selected genes were further examined (Additional file 8: Figure S7). Favorable alleles of 18 selected genes increased significantly from CL to MCC over different decades, indicating that they were positively selected from landraces to modern cultivars (Fig. 3a, b). These included six higher grain yield-related alleles (*Sus1-7B-Hap-T*, *Sus2-2A-Hap-A*, *GW2-6A-Hap-A*, *GW2-6B-Hap-1*, *CKX-D1a* and *TKW6-A1a*), six great processing and end-use quality alleles (*Glu-D1d*, *Pina-D1b*, *Pinb-D1b*, *Pds-B1b*, *Pod-A1b* and *Wx-B1b*), and six stress resistance factors (*Lr14+*, *Lr34+*, *Yr15+*, *VP-1Bc*, *1BL.1RS* and *Dreb-D1a*) (Additional file 8: Figure S7). In contrast, the frequencies of favorable alleles at 14 loci decreased from CL to MCC (Fig. 3c, d). This might have been caused by negative selection during breeding and therefore indicates underutilization in modern breeding. The 14 alleles included four for grain yield (*GASR-A1-H1c*, *Cwi-4A-Hap-C*, *GS-D1a* and *MOC-7A-Hap-H*), five for quality (Glu-A1- Ax1 or Ax2*, *Glu-B1-7OE*, *Pinb-B2b*, *Lcy-B1b* and *Zds-A1a*), and five for stress tolerance (*MFT-A1-PHS+*, *Sdr-B1a*, *1-fehw3-Westonia type*, *PHS1-PHS+* and *Fhb1+*) (Additional file 8: Figure S7). Additionally, *Rht-B1b*, *Rht-D1b*, *vrn-D1* and *Ppd-D1a* predominated in modern Chinese cultivars at adaptation. Allelic variations of the remaining 11 genes, including *vrn-A1*, *Cwi-5D-Hap-C*, *Sus1-7A-Hap-H*, *Psy-A1b*, *Psy-B1a or b* and *Psy-D1a*, were present at high frequencies in both CL and MCC, suggesting that they were nearly fixed before wheat improvement (Fig. 3e, f; Additional file 8: Figure S7). In contrast, *Lr68+*, *Ppo-A1b*, *Ppo-D1a*, and *Sus2-2B-Hap-H* existed at low frequencies in both subgroups and could be considered as the further major improvement targets in breeding (Fig. 3g, h; Additional file 8: Figure S7).

**Fig. 4:**
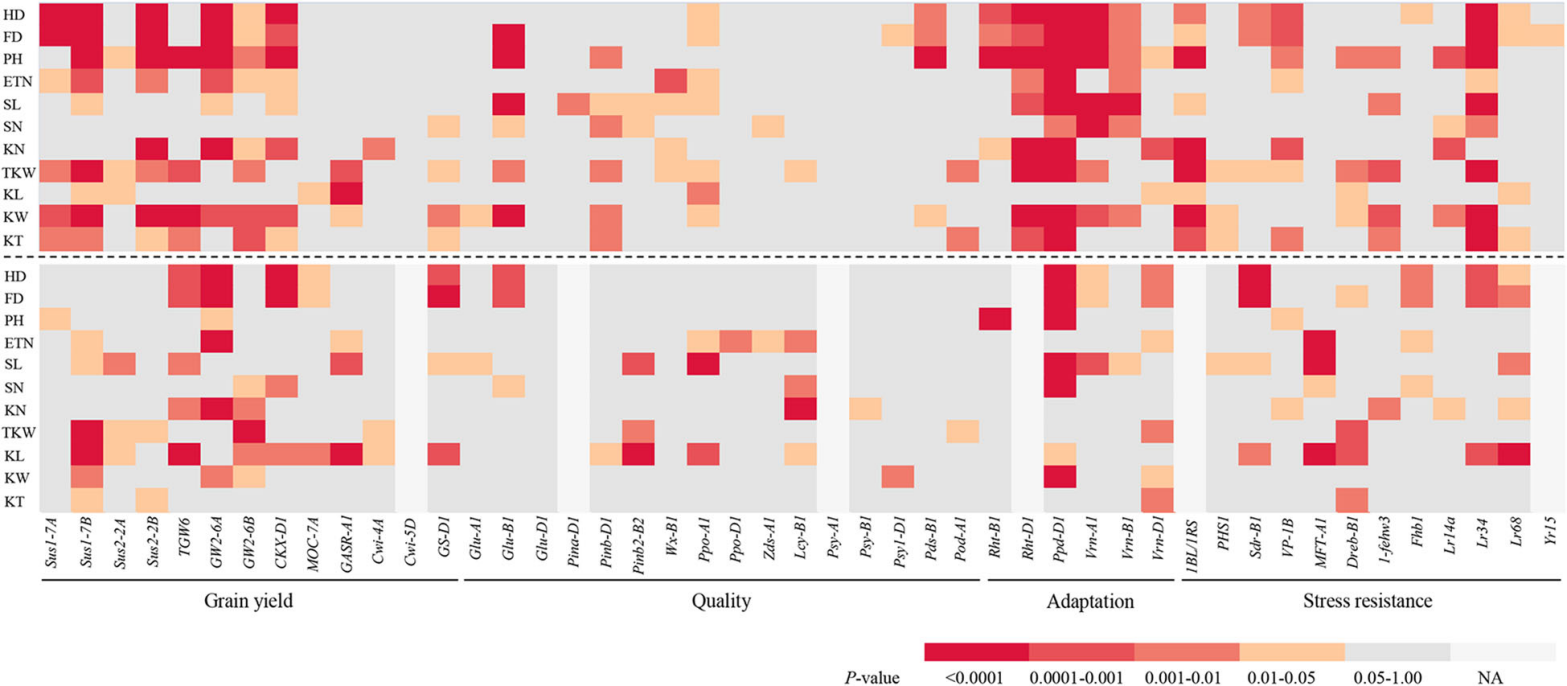
Heat maps of significant signals of 47 genes associated with eleven agronomic traits in Chinese landraces (CL) and modern Chinese cultivars (MCC). Significance levels of loci associated with agronomic traits in the MCC are shown above horizontal dashed lines and those for CL are below. Association signals from significant to weak are indicated by different colors from red to light gray. HD: heading date; FD: flowering date; PH: plant height; ETN: effective tiller number; SL: spike length; SN: spikelet number per spike; KN: kernel number per spike; TKW: 1000-kernel weight; KL: kernel length; KW: kernel width; KT: kernel thickness

To further dissect the selected alleles we used ANOVA of eleven traits to identify association signals in both landraces and modern cultivars based on population structure analyses above. A total of 189 association signals in modern cultivars and 133 association signals for landraces were evaluated with a significant threshold of *P*<0.05. More association signals were detected in modern cultivars than in landraces (Fig. 4; Additional file 1: Table S5). We identified more association signals for the trait TKW in modern cultivars compared with other traits, consistent with the notion that high yield was the main goal of breeding. To further confirm selected loci corresponding to the target traits we compared the association signals and selective signatures in the 47 functional genes. Nine selected loci underlaid the corresponding traits in modern cultivars or landraces (Fig. 2). Three adaptation genes, *Rht-B1*, *Rht-D1* and *Ppd-D1*, accounted for 7–46% of the phenotypic variances explained by PH, HD, and FD. Six grain-yield genes were responsible for 1–7% of the phenotypic variances explaining their target traits (Additional file 1: Table S4). In addition, some genes were associated not only with the target traits but also other traits, indicating that these genes had pleiotropic genetic effects (Fig. 4; Additional file 1: Table S5).

### Global status of 47 functional genes in wheat

**Fig. 5:**
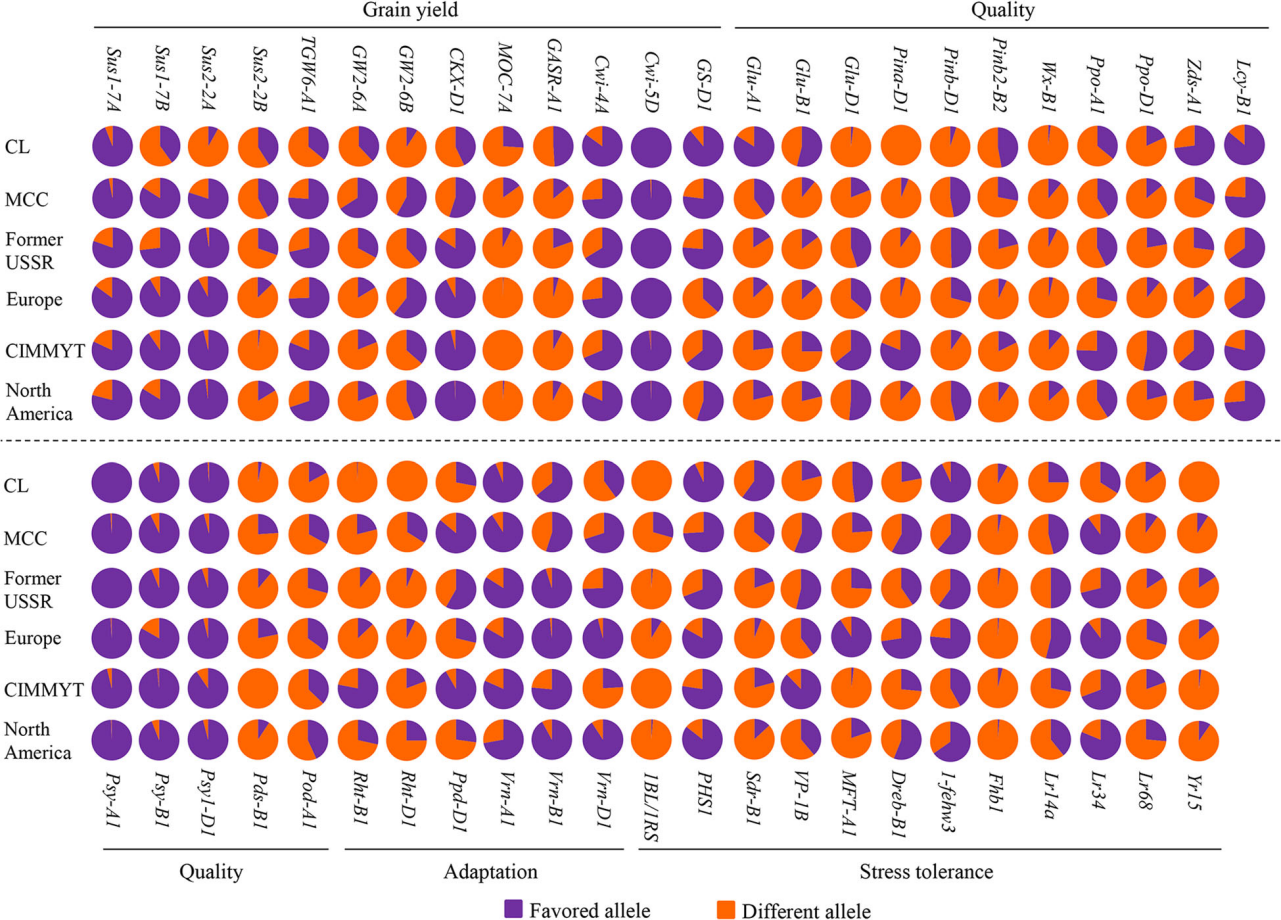
Allele frequencies of 47 genes controlling grain yield, quality, adaptation and stress resistance among subgroups, i.e. North America, CIMMYT, Europe, the former USSR, and China. According to breeding objectives for high yield, great quality, stress resistance and adaptation, the alleles that suffered strong selections in this study were favorable alleles influencing high thousand kernel weight, excellent quality, stress resistance, and widely adaptation

Allelic variations of the 47 genes were evaluated according to geographic distributions (Fig. 5). Alleles at eight loci including five associated with higher TKW (*Sus1-7A-Hap-H*, *Sus1-7B-Hap-T*, *Sus2-2A-Hap-A*, *TGW6-A1a* and *Cwi-4A-Hap-C*) and three for winterness (*vrn-A1*), leaf rust resistance (*Lr34+*) and low pre-harvest sprouting (*PHS1-PHS+*) predominated in all modern cultivars, indicating strong selection pressure. Favorable allelic frequencies of *Psy-A1b*, *Psy-B1a or b*, *Psy-D1a* and *Cwi-5D-Hap-C* approached almost 100% and were fixation before improvement selection. In contrast, all modern cultivars possessed lower frequency of the favorable alleles in nine loci, including two for higher grain yield (*MOC-7A-Hap-H* and *GASR-A1-H1c*), four for great grain quality (*Glu-B1-7OE*, *Pinb-B2b*, *Wx-B1b* and *Pod-A1b*), and three for disease resistance (*Lr68+*, *Fhb1+* and *Yr15+*), indicating the underutilization of these genes in breeding. Alleles associated with hard grain texture (*Pina-D1b* and *Pinb-D1b*), high molecular weight glutenin subunit (*Glu-D1d*), lower yellow pigment content (*Pds-B1b*), reduction of plant height (*Rht-B1b* and *Rht-D1b*), and 1BL/1RS translocation were rare or absent in CL but present in the MCC and introduced cultivars, suggesting these genes might originate from foreign countries, and played an important role in Chinese wheat improvement.

### Introduced cultivars made a significant genetic contribution to modern Chinese cultivars

**Fig. 6:**
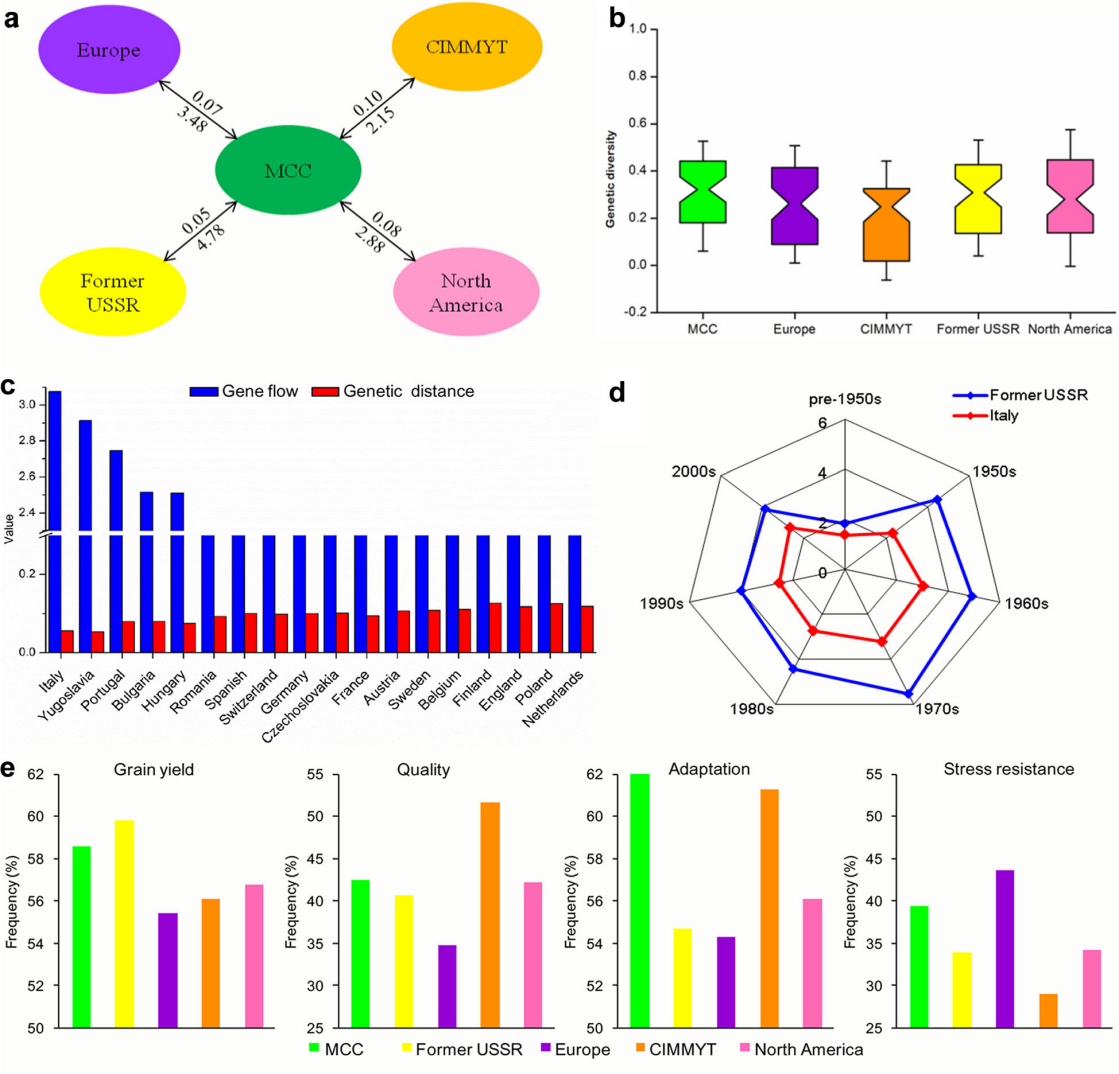
Comparison of genetic variation and divergence in wheat cultivars worldwide. **a** Genetic differentiation coefficients (*Fst*) and gene flows between subgroups are shown above and below the joining lines. **b** Genetic diversity of modern Chinese cultivars (MCC) and introduced cultivars (IC) from four regions. **c** Gene flow and genetic distance between MCC and European accessions composed of varieties from 18 countries. **d** Gene flow between MCC and accessions from the former USSR and Italy during different decades. **e** Comparison of average favorable allele frequencies of genes controlling grain yield, quality, adaptation and stress resistance in different regions. According to breeding objectives for high yield, good quality, stress resistance and adaptation, the alleles that suffered strong selections in this study were favorable alleles influencing high thousand kernel weight, excellent quality, stress resistance, and widely adaptation

To evaluate the contribution of introduced cultivars from different geographic regions to modern Chinese cultivars we measured gene flow and allele differences. Gene flow was frequent (4.78) and genetic differentiation was low (0.05) in comparing the MCC with the former USSR (Fig. 6a). Similarly, the index of genetic diversity of each subgroup ranged from 0.26 (CIMMYT) to 0.32 (MCC) (Fig. 6b). Further analysis between the MCC and accessions from 18 European countries showed that the MCC had the highest gene flow (3.08) involving accessions from Italy (Fig. 6c). Although gene flows between the former USSR, Italy and the MCC during the 1950s to 1970s were high, it declined after the 1970s (Fig. 6d).

We compared the average allele number at 47 genes in different regions, in order to evaluate the effect of introduced cultivars (Additional file 9: Figure S8a). The favorable allele number in modern Chinese cultivars was considerably higher than average, and has been increasing since the 1970s, when the “Green Revolution” swept through China. The use of semi-dwarf wheat is very widespread in China as elsewhere. The frequency of *Rht-D1b* in the MCC was higher than in other regions, but *Rht-B1b* occurred at a relatively low frequency in MCC comparing with CIMMYT because of the use of different dwarf germplasms between modern Chinese cultivars and introduced cultivars (Additional file 9: Figure S8b). The average allele frequencies of genes controlling four kinds of traits were compared in different subgroups (Fig. 6e). The MCC had higher favorable allelic frequencies than IC for grain yield. For example, alleles controlling higher grain weight including *Sus1-7A-Hap-H*, *Sus2-2B-Hap-H*, and *GW2-6A-Hap-A* were present at high frequencies in modern Chinese cultivars (Fig. 5). Besides, photoperiod-insensitive type (*Ppd-D1a*) predominated in cultivars from China, CIMMYT and the former USSR. However, the variability at various loci affecting quality indicated a greater influence on CIMMYT than other regions in breeding for end-use quality traits (Fig. 6e). These alleles influenced hard grain texture (*Pina-D1b*), low PPO activity (*Ppo-A1b* and *Ppo-D1a*) and lower yellow pigment content (*Zds-A1a*) (Fig. 5). For stress resistance, the average favorable allele frequency predominated in Europe compared with other regions, such as two genes governing drought tolerance (*Dreb-B1a* and *1-fehw3-Westonia type*), one for low pre-harvest sprouting (*MFT-A1-PHS+*) and one for leaf rust resistance (*Lr68+*) (Fig. 5). Hence, those introduced germplasms with numerous beneficial traits are vital for improving inferior characteristics in modern cultivars and played a fundamental role in broadening the base of wheat breeding in China.

## Discussion

### Excellent landraces form the basis of early modern Chinese wheat breeding

Landraces have played an important role in early Chinese breeding programs [9]. In this study, we showed that both genetic distance and population differentiation were lower between landraces and modern cultivars in the pre-1950s compared with that during other decades (Additional file 7: Figure S6c). In addition, genetic diversity was lost from landraces (0.26) to modern cultivars (0.24) because a limited number of superior landraces were used in initial breeding programs (Additional file 7: Figure S6b). Looking back on the history of Chinese wheat breeding, we know that in the early 1950s large numbers of landraces were collected and evaluated in different ecological regions, and the highest-ranking landraces with better yield potential and disease tolerance were directly adopted for production [42]. A small population bottleneck occurred when these landraces were extensively used in breeding. Nevertheless, with the development of genotyping techniques, mining favorable allelic variations at genes hidden in landraces, such as the low-frequency alleles detected in the current study, including *Lr68+*, *Ppo-D1a* and *Fhb1+*, will contribute to wheat breeding (Additional file 8: Figure S7). In addition, an objective appraisal should be made to ensure that KASP markers for functional genes are developed from known polymorphic sites by sequencing several genetically divergent samples keeping in mind the possibility of flaws such as ascertainment bias. It is possible that landraces may have rare or novel mutations in alleles that could not be identified by the KASP markers. With the availability of the complete reference genome of Chinese Spring, coupled with on-going international efforts to sequence the wheat pan-genome [2, 43], it leads to be possible to mine more genetic diversity through high-throughput and cost-effective sequencing techniques [11].

### Introduced cultivars were important components of modern Chinese cultivars especially prior to 1980

Introduced varieties pushed Chinese breeding history forward since the mid-1950s, and some of them were founder parents [9]. In this study, genetic diversity was similar between modern Chinese cultivars (0.32) and introduced cultivars (0.29), but they were significantly higher than that of the landraces (0.26) at the gene level. This indicates that worldwide breeding has not reduced the overall genetic diversity of wheat [3], which was similar to maize [44] and rice [18]. Furthermore, higher gene flow values for the MCC with either former USSR (4.78) or Italy (3.08) indicated that introduced cultivars especially from these two regions contributed greatly to the genetic variations of modern Chinese cultivars (Fig. 6a). These results are consistent with historical reports that Italian varieties Villa Glori, Mentana, Funo, Abbondanza, St 2422/464 and Libellula performed very well in the Yellow and Huai River valley winter wheat region (Zone II), lower and middle Yangtze River valley winter wheat region (Zone III), southwestern winter wheat region (Zone IV), and northwestern spring wheat region (Zone VIII). Varieties such as Ukraine 0246, New Ukraine, Red Star, Kavkaz and Aurora from the former USSR were disseminated mostly in Xinjiang [42]. Other counties contributed to Chinese wheat breeding, but to a lesser degree. For example, CIMMYT wheats with low sensitivity to the photoperiod are widely adapted to diverse climatic conditions [45]. We found that *Ppd-D1a* conferring photoperiod insensitivity had a higher frequency in both MCC (89%) and CIMMYT (92%) (Fig. 5), indicating that the germplasm originating from CIMMYT probably exerted a certain function in conferring on Chinese wheat wide adaptation. In addition, we found good grain quality in CIMMYT wheats and stress resistance from Europe germplasms (Fig. 6e). Overall, our results support the point that the Chinese wheat improvement was based on hybridization programs involving well-adapted landraces and introduced accessions. Thus worldwide germplasm exchange and utilization is an established way to widen the genetic basis in wheat breeding.

### Selection signals implicate the direction of molecular breeding of wheat

Selection during breeding drastically reshapes crop genomes, which accumulates beneficial alleles located in both genic and non-genic regions [46]. In the current study, we found that favorable alleles were present in higher proportions in modern cultivars than in landraces due to the endeavors of breeders. This supports the fact that artificial selection eliminates undesirable or even deleterious alleles, and may cause accumulation of potentially valuable alleles for the future demands of producers and consumers [46, 47]. The detection of loci being selected during crop improvement contributes to more targeted breeding efforts and provides opportunities to improve genomic selection models [14]. The effects on the remaining genetic diversity might not only be positive, but also could be negative or neutral [21]. In this study, we identified selected alleles that originated from landraces and introduced materials (Fig. 5). Alleles *Sus1-7A-Hap-H*, *Cwi-5D-Hap-C*, *Psy-A1b*, *Psy-B1a or b*, *Psy-D1a*, and *vrn-A1* had high frequencies in both landraces and modern cultivars (Additional file 8: Figure S7), indicating that they were almost fixed before modern wheat breeding. We also found low frequencies of favorable alleles at *Lr68*, *Ppo-D1*, *Ppo-A1*, *Sus2-2B* existed in both subgroups, where selection could be focused in the future.

In summary, 18 favorable and 14 unfavorable alleles influencing grain yield, quality and stress resistance were strongly selected by comparing allele frequencies using Z tests (Fig. 2a). Of the 13 loci affecting yield, favorable alleles at *Sus1-7B*, *Sus2-2A*, *GW2-6B*, *TGW6-A1* and *GW2-6A* were subjected to strong selection, and *Cwi-5D-Hap-C* was almost fixed. For end use quality, overwhelmingly strong selection had occurred before intensive breeding at *Psy-A1b*, *Psy-B1a* or *b* and *Psy-D1a* because of the global desire for white flour products. For resistance to pre-harvest sprouting, *PHS1-PHS+* was strongly selected. *Lr34+* was global favored due to its broad-spectrum resistance (Fig. 5). At the important loci controlling adaptation, the strongest selection was for *vrn-A1*, the winter allele that was almost global fixed, and variations were mainly found at *Vrn-B1* and *Vrn-D1*. The photoperiod insensitive allele *Ppd-D1a* was strongly favored in China, CIMMYT and the former USSR. Allelic variations of *Pina-D1b*, *Pinb-D1b*, *Glu-D1d*, *Pds-B1b*, *Rht-B1b*, *Rht-D1b* and *1BL.1RS* were rare or absent in Chinese landraces but present in both modern Chinese cultivars and introduced cultivars. Importantly, *Lr68+*, *Fhb1+*, *Wx-B1b* and *Yr15+* existed at a low frequency for all accessions, which could be regarded as improvement targets in global wheat breeding.

### Crucial functional genes are often pleiotropic

In the present study, we identified genes controlling adaptation, stress resistance, grain quality, and yield-related traits during wheat improvement. In common with a report based on a 9 K SNP chip analysis of wheat worldwide [3], we detected the same loci selected in breeding, including the well-known green revolution genes *Rht-B1* and *Rht-D1* and flowering time regulation genes *Vrn-D1* and *Ppd-D1*. Moreover, almost all markers from genes *Sus1-7B*, *GW2-6A*, *GW2-6B*, *CKX-D1*, *GASR-A1* and *GS-D1* regulating yield-related traits were associated with their target traits (Fig. 4; Additional file 1: Table S4).

Association analysis showed that a number of selected loci not only influenced the target trait but also other traits (Additional file 1: Table S4). For example, the application of the *Rht-B1* and *Rht-D1* semi-dwarfing genes led to significant increases in wheat yields [5]. Numerous reports have shown that quantitative trait loci (QTL) for kernel number per spike and thousand-kernel weight were associated with *Rht-B1* and *Rht-D1* [48–51]. In our study, *Rht-B1* and *Rht-D1* not only influenced plant height (PH) but also heading date (HD), flowering date (FD), kernel number per spike (KN), effective tiller number (ETN), spike length (SL), and 1000-kernel weight (TKW) (Additional file 1: Table S4). In wheat, the decrease in stem elongation caused by GA-insensitivity results in improved resistance to stem lodging and increased in assimilate partitioning to developing ears, enabling greater floret survival at anthesis and increased grain numbers per ear [52, 53]. We found that more traits were associated with *Rht-D1* than with *Rht-B1* (Fig. 4; Additional file 1: Table S5) because of different functional elements in promoter regions, and thus potentially different patterns of expression [54]. Combined with the complex network of interactions that modify the action of DELLA proteins, it was not difficult to determine that the dwarf-type *Rht-B1b* and *Rht-D1b* DELLA proteins affect different aspects of plant phenotype [51]. In the examples (*Rht-B1*, *Rht-D1* and *Ppd-D1*) examined in wheat, these important genes have pleiotropic effects [55, 56]. We found that *Ppd-D1*, as a photoperiod gene for heading date (HD) and flowering date (FD), also greatly influenced plant height (PH), kernel number per spike (KN), effective tiller number (ETN), spike length (SL), 1000-kernel weight (TKW), kernel length (KL), kernel width (KW) and kernel thickness (KT) (Additional file 1: Table S4). The *Ppd-D1* gene not only confers photoperiod insensitivity but also enhances yield potential, which means that *Ppd-D1* promotes heading and maturation, reduces tiller, plant height, and kernel number per spike [57, 58]. The effect is possibly associated with increased early resource capture [56] and increased spikelet fertility to influence the shortened growth period and reduced tiller and spikelet number. In addition, some QTLs controlling yield traits located on chromosomes 2A, 2B and 2D were also associated with photoperiod insensitivity generally [59]. Therefore, crucial functional genes with pleiotropic effects would provide important information on dissecting genetic bases of wheat improvement.

### Cross breeding promoted gene flow among populations and improved the diversity of functional genes in the MCC

Modern wheat breeding reflects the most important targets of artificial selection and creates superior plant genotypes and phenotypes that are fixed in cultivars with improved yield, stability, nutritional qualities, and other commercial traits [8, 21]. Forty seven genes controlling grain yield, quality, adaptation, and stress resistance were included in this study. Based on KASP markers developed from these important genes, we found that selection caused genetic diversity increasing for functional genes (Additional file 7: Figure S6a). On the one hand, modern wheat breeding programs have aimed to develop high-yield varieties, and thus favored adaptation and yield-related alleles, such as those selected for photoperiod, vernalization, and grain yield, resulting in previously rare alleles becoming frequent in modern cultivars [21, 60]. On the other hand, modern plant breeding has promoted gene exchanges and recombination in the gene coding regions for self-pollinated wheat [22]. Hence, cross breeding has increased genetic diversity of modern cultivars in comparison to landraces. In addition, introgression of novel germplasms in wheat breeding could be beneficial for averting the narrowing of genetic diversity.

## Conclusions

Genetic variation and phenotypic differences were analyzed using representative 1152 wheat accessions from Asia, Europe, North America and CIMMYT based on KASP assays of 47 genes controlling grain yield, quality, adaptation and stress resistance. Selection signals and gene flow indicated that mining novel alleles of landraces and utilizing introduced accessions are effective ways to improve modern varieties. This study objectively reports the distribution of important genes in global wheats and may be useful for wheat molecular breeding.

## Additional files

Additional file 1: **Table S1.** Detailed information on materials and their allelic variations of 47 genes used in this study. **Table S2.** Basic information including allelic variations and primer sequences for the 52 KASP assays. **Table S3.** Basic descriptive statistics in Chinese landraces (CL) and modern Chinese cultivars (MCC) for eleven traits using BLUP values. **Table S4.** Putative selective genes during wheat improvement based on the BLUP values of phenotypic traits. **Table S5.** Genome-wide association signals of 47 genes controlling adaptation and yield-related traits. (XLS 1118 kb)

Additional file 2: **Figure S1.** Geographic origins of 1152 global wheat accessions used in the current study. **a** Geographic origins of Chinese wheat cultivars. Green shows modern cultivars; Red means landraces. The map of China is available at http://bzdt.nasg.gov.cn/. **b** The number of accessions in different subgroups. (PDF 2082 kb)

Additional file 3: **Figure S2.** Analysis of population structure in 480 Chinese wheat accessions based on 47 KASP markers. **a** Neighbor-joining tree of 323 modern Chinese cultivars and 157 Chinese landraces. Green and red lines represent modern Chinese cultivars and landraces, respectively. **b** PCA plots of 480 accessions based on the same markers. (PDF 2362 kb)

Additional file 4: **Figure S3.** Analysis of population structure in 672 introduced cultivars based on 47 KASP markers. **a** Neighbor-joining tree of 672 introduced accessions from four regions, including Europe, CIMMYT, the former Soviet Union (former USSR) and North America, marked in purple, green, yellow and pink, respectively. **b** PCA plots of 672 accessions based on the same markers. (PDF 2647 kb)

Additional file 5: **Figure S4.** Phenotypic variations of 11 agronomic traits in Chinese landraces (CL) and modern Chinese cultivars (MCC). Phenotypic values of CL and MCC are indicated by red histograms with a dashed black line and green histograms with a solid black line, respectively. HD: heading date (days); FD: flowering date (days); PH: plant height (cm); ETN: effective tiller number (number); SL: spike length (cm); SN: spikelet number per spike (number); KN: kernel number per spike (number); TKW: 1000-kernel weight (g); KL: kernel length (mm); KW: kernel width (mm); KT: kernel thickness (mm). (PDF 1928 kb)

Additional file 6: **Figure S5.** Frequency distribution of phenotypic variations of 11 agronomic traits in Chinese landraces (CL) and modern Chinese cultivars (MCC) in three environments. Phenotypic values of CL and MCC are indicated by a red histogram with a dashed black line and a green histogram with a solid black line, respectively; 2014, 2015, and 2016 indicate the years in which all accessions were planted. **a** HD: heading date (days); **b** FD: flowering date (days); **c** PH: plant height (cm); **d** ETN: effective tiller number (number); **e** SL: spike length (cm); **f** SN: spikelet number per spike (number); **g** KN: kernel number per spike (number); **h** TKW: 1000-kernel weight (g); **i** KL: kernel length (mm); **j** KW: kernel width (mm); **k** KT: kernel thickness (mm). (PDF 5754 kb)

Additional file 7: **Figure S6.** Genetic diversity and population differentiation of Chinese landraces (CL) and modern Chinese cultivars (MCC). **a** Genetic diversity between CL and MCC. **b** Genetic diversity of the MCC during different decades. **c** Genetic differentiation and distance between CL and MCC during different decades. *Fst* and genetic distance are marked in red and blue, respectively. (PDF 2136 kb)

Additional file 8: **Figure S7.** Allele frequencies of 47 loci in Chinese landraces (CL) and modern Chinese cultivars (MCC). Red histogram shows CL, green histogram means MCC. **a, c, e** and **g** show allele frequencies in CL and MCC according to traits. **b, d, f** and **h** show changes in allele frequencies during different decades. *P<0.05; **P<0.01; ***P<0.001; NS, not significant. (PDF 8038 kb)

Additional file 9: **Figure S8.** Comparison of the numbers and frequencies of favorable alleles in global wheat accessions. **a** Bean plot for the distribution of favorable allele numbers in introduced cultivars (IC) from four regions and modern Chinese cultivars (MCC) during different decades. **b** Allele frequencies of *Rht-B1* and *Rht-D1* in different groups worldwide. (PDF 1151 kb)

## Abbreviations

BLUP: Best linear unbiased predictor
CIMMYT: International Maize and Wheat Improvement Center
CL: Chinese landraces
CTAB: Cetyl Trimethyl Ammonium Bromide
ddH_2_O: Double distilled water
DNA: Deoxyribonucleic acid
ETN: Effective tiller number
FD: Flowering date
Fst: Fixation index
HD: Heading date
InDel: Insertion or Deletion
KASP: Kompetitive Allele Specific PCR
KL: Kernel length
KN: Kernel number per spike
KT: Kernel thickness
KW: Kernel width
LGC: Laboratory of the Government Chemist
MCC: Modern Chinese cultivars
NTC: Non-template control
PCA: Principal component analysis
PH: Plant height
SL: Spike length
SN: Spikelet number per spike
SNP: Single Nucleotide Polymorphism
TKW: 1000-kernel weight

## References

[1] Dubcovsky J, Dvorak J (2007). Genome plasticity a key factor in the success of polyploid wheat under domestication. Science.

[2] Brenchley R, Spannagl M, Pfeifer M, Barker GL, D’Amore R, M. Allen A, McKenzie N, Kramer M, Kerhornou A, Bolser D, Kay S, Waite D, Trick M, Bancroft L, Gu Y, Huo N, Luo MC, Sehgal S, Kianian S, Gill B, Anderson O, Kersey P, Dvorak J, McCombie WR, Hall A, Mayer KF, J. Edwards K, W. Bevan M, Hall N. Analysis of the bread wheat genome using whole-genome shotgun sequencing. Nature 2012;491(7426):705–710. 10.1038/nature11650 PMC3510651 23192148

[3] Cavanagh CR, Chao SM, Wang SC, Huang BE, Stephen S, Kiani S, Forrest K, Saintenac C, Brown-Guedira GL, Akhunova A, See D, Bai GH, Pumphrey M, Tomar L, Wong DB, Kong S, Reynolds M, da Silva ML, Bockelman H, Talbert L, Anderson JA, Dreisigacker S, Baenziger S, Carter A, Korzun V, Morrell PL, Dubcovsky J, Morell MK, Sorrells ME, Hayden MJ, Akhunov E (2013). Genome-wide comparative diversity uncovers multiple targets of selection for improvement in hexaploid wheat landraces and cultivars. Proc Natl Acad Sci.

[4] Yamasaki M, Wright SI, Mcmullen MD (2007). Genomic screening for artificial selection during domestication and improvement in maize. Ann Bot.

[5] Hedden P (2003). The genes of the green revolution. Trends Genet.

[6] Worland T, Snape JW, Bonjean AP, Angus WJ (2001). Genetic basis of worldwide varietal improvement. The world wheat book: a history of wheat breeding.

[7] Reynolds M, Dreccer F, Trethowan R (2007). Drought-adaptive traits derived from wheat wild relatives and landraces. J Exp Bot.

[8] Moose SP, Mumm RH (2008). Molecular plant breeding as the foundation for 21st century crop improvement. Plant Physiol.

[9] Zhuang QS (2003). Chinese wheat improvement and pedigree analysis.

[10] Lopes MS, El-Basyoni I, Baenziger PS, Singh S, Royo C, Ozbek K, Aktas H, Ozer E, Ozdemir F, Manickavelu A, Ban T, Vikram P (2015). Exploiting genetic diversity from landraces in wheat breeding for adaptation to climate change. J Exp Bot.

[11] Rasheed A, Mujeeb-Kazi A, Ogbonnaya FC, He ZH, Rajaram S (2018). Wheat genetic resources in post-genomics era: promise and challenges. Ann Bot.

[12] Li XJ, Xu X, Liu WH, Li XQ, Yang XM, Li LH (2009). Genetic contribution of introduced varieties to wheat breeding in China evaluated using SSR markers. Acta Agron Sin.

[13] Heffner EL, Sorrells ME, Jannink JL (2009). Genomic selection for crop improvement. Crop Sci.

[14] Morrell PL, Buckler ES, Ross-Ibarra J (2012). Crop genomics: advances and applications. Nat Rev Genet.

[15] Zhou ZK, Jiang Y, Wang Z, Gou ZH, Lyu J, Li WY, Yu YJ, Shu LP, Zhao YJ, Ma YM, Fang C, Shen YT, Liu TF, Li CC, Li Q, Wu M, Wang M, Wu YS, Dong Y, Wan WT, Wang X, Ding ZL, Gao YD, Xiang H, Zhu BG, Lee SH, Wang W, Tian ZX (2015). Resequencing 302 wild and cultivated accessions identifies genes related to domestication and improvement in soybean. Nat Biotechnol.

[16] Fang L, Wang Q, Hu Y, Jia YH, Chen JD, Liu BL, Zhang ZY, Guan XY, Chen SQ, Zhou BL, Mei GF, Sun JL, Pan ZE, He SP, Xiao SH, Shi WJ, Gong WF, Liu JG, Ma J, Cai CP, Zhu XF, Guo WZ, Du XM, Zhang TZ (2017). Genomic analyses in cotton identify signatures of selection and loci associated with fiber quality and yield traits. Nat Genet.

[17] Huang XH, Kurata N, Wei XH, Wang ZH, Wang AH, Zhao Q, Zhao Y, Liu KY, Lu HY, Li WJ, Guo YL, Lu YQ, Zhou CC, Fan DL, Weng QJ, Zhu CR, Huang T, Zhang L, Wang YC, Feng L, Furuumi H, Kubo T, Miyabayashi T, Yuan XP, Xu Q, Dong GJ, Zhan QL, Li CY, Fujiyama A, Toyoda A, Lu TT, Feng Q, Qian Q, Li JY, Han B (2012). A map of rice genome variation reveals the origin of cultivated rice. Nature.

[18] Xie WB, Wang GW, Yuan M, Yao W, Lyu K, Zhao H, Yang M, Li PB, Zhang X, Wang QX, Liu F, Dong HX, Zhang LJ, Li XL, Meng XZ, Zhang W, Xiong LZ, Wang SP, Yu SB, Xu CO, Luo J, Li HX, Xiao JH, Lian XM, Zhang QF (2015). Breeding signatures of rice improvement revealed by a genome variation map from a large germplasm collection. Proc Natl Acad Sci.

[19] Hufford MB, Xu X, van Heerwaarden J, Pyhäjärvi T, Chia JM, Cartwright RA, Elshire RJ, Glaubitz JC, Guill KE, Kaeppler SM, Lai JS, Morrell PL, Shannon LM, Song C, Springer NM, Swanson-Wagner RA, Tiffin P, Wang J, Zhang GY, Doebley J, McMullen MD, Ware D, Buckler ES, Yang S, Ross-Ibarra J (2012). Comparative population genomics of maize domestication and improvement. Nat Genet.

[20] Wang SC, Wong D, Forrest K, Allen A, Chao S, Huang BE, Maccaferri M, Salvi S, Milner SG, Cattivelli L, Mastrangelo AM, Whan A, Stephen S, Barker G, Wieseke R, Plieske J, Lillemo M, Mather D, Appels R, Dolferus R, Brown-Guedira G, Korol A, Akhunova AR, Feuillet C, Salse J, Morgante M, Pozniak C, Luo MC, Dvorak J, Morell M, Dubcovsky J, Ganal M, Tuberosa R, Lawley C, Mikoulitch I, Cavanagh C, Edwards KJ, Hayden M, Akhunov E, International Wheat Genome Sequencing Consortium (2014). Characterization of polyploid wheat genomic diversity using a high-density 90,000 single nucleotide polymorphism array. Plant Biotechnol J.

[21] Gao LF, Zhao GY, Huang DW, Jia JZ (2017). Candidate loci involved in domestication and improvement detected by a published 90K wheat SNP array. Sci Rep.

[22] Hao CY, Wang YQ, Chao SM, Li T, Liu HX, Wang LF, Zhang XY (2017). The iSelect 9K SNP analysis revealed polyploidization induced revolutionary changes and intense human selection causing strong haplotype blocks in wheat. Sci Rep.

[23] Rasheed A, Hao YF, Xia XC, Khan A, Xu YB, Varshney RK, He ZH (2017). Crop breeding chips and genotyping platforms: progress, challenges, and perspectives. Mol Plant.

[24] Yamasaki M, Tenaillon MI, Bi IV, Schroeder SG, Sanchez-Villeda H, Doebley JF, Gaut BS, McMullen MD. A large-scale screen for artificial selection in maize identifies candidate agronomic loci for domestication and crop improvement. Plant Cell 2005;17(11):2859–2872. 10.1105/tpc.105.037242 PMC1276015 16227451

[25] Liu Y, He ZH, Appels R, Xia XC (2012). Functional markers in wheat: current status and future prospects. Theor Appl Genet.

[26] Rasheed A, Wen WE, Gao FM, Zhai SN, Jin H, Liu JD, Guo Q, Zhang YJ, Dreisigacker S, Xia XC, He ZH (2016). Development and validation of KASP assays for genes underpinning key economic traits in bread wheat. Theor Appl Genet.

[27] Semagn K, Babu R, Hearne S, Olsen M (2014). Single nucleotide polymorphism genotyping using Kompetitive allele specific PCR (KASP): overview of the technology and its application in crop improvement. Mol Breed.

[28] Hao CY, Dong YS, Wang LF, You GX, Zhang HN, Ge HM, Jia JZ, Zhang XY (2008). Genetic diversity and construction of core collection in Chinese wheat genetic resources. Chin Sci Bull.

[29] Chen DH (1999). Ronald PC. A rapid DNA minipreparation method suitable for AFLP and other PCR applications. Plant Mol Biol Report.

[30] Bernardo R (1996). Best linear unbiased prediction of maize single cross performance. Crop Sci.

[31] Pielou EC (1969). An introduction to mathematical ecology.

[32] Nei M, Tajima F, Tateno Y (1983). Accuracy of estimated phylogenetic trees from molecular data. II Gene frequency data J Mol Evol.

[33] Liu K, Muse SV (2005). PowerMarker: an integrated analysis environment for genetic marker analysis. Bioinformatics.

[34] Tamura K, Peterson D, Peterson N, Stecher G, Nei M, Kumar S (2011). MEGA5: molecular evolutionary genetics analysis using maximum likelihood, evolutionary distance, and maximum parsimony methods. Mol Biol Evol.

[35] Jombart T, Devillard S, Balloux F (2010). Discriminant analysis of principal components: a new method for the analysis of genetically structured populations. BMC Genet.

[36] Hudson RR, Slatkin M, Maddison WP (1992). Estimation of levels of gene flow from DNA sequence data. Genetics.

[37] Slatkin M, Barton NH (1989). A comparison of three indirect methods for estimating average levels of gene flow. Evolution.

[38] Yeh FC, Yang RC, Boyle TB, Ye ZH, Mao JX, Yeh C, Timothy B, Mao X (1999). Popgene version 1.32: the user friendly software for population genetic analysis. Molecular biology and biotechnology Centre.

[39] Doebley JF, Gaut BS, Smith BD (2006). The molecular genetics of crop domestication. Cell.

[40] Qiu Q, Wang LZ, Wang K, Yang YZ, Ma T, Wang ZF, Zhang X, Ni ZQ, Hou FJ, Long RJ, Abbott R, Lenstra J, Liu JQ (2015). Yak whole-genome resequencing reveals domestication signatures and prehistoric population expansions. Nat Commun.

[41] Li YH, Zhao SC, Ma JX, Li D, Yan L, Li J, Qi XT, Guo XS, Zhang L, He WM, Chang RZ, Liang QS, Guo Y, Ye C, Wang XB, Tao Y, Guan RX, Wang JY, Liu YL, Jin LG, Zhang XQ, Liu ZX, Zhang LJ, Chen J, Wang KJ, Nielsen R, Li RQ, Chen PY, Li WB, Reif JC, Purugganan M, Wang J, Zhang MC, Wang J, Qiu LJ (2013). Molecular footprints of domestication and improvement in soybean revealed by whole genome re-sequencing. BMC Genomics.

[42] He ZH, Rajaram S, Xin ZY, Huang GZA (2001). History of wheat breeding in China.

[43] International Wheat Genome Sequencing Consortium (IWGSC) (2014). A chromosome-based draft sequence of the hexaploid bread wheat (*Triticum aestivum* L.) genome. Science.

[44] van Heerwaarden J, Hufford MB, Ross-Ibarra J (2012). Historical genomics of north American maize. Proc Natl Acad Sci.

[45] Syme JR (1968). Ear emergence of Australian, Mexican and European wheats in relation to time of sowing and their response to vernalization and day length. Aust J Exp Agric.

[46] Shi JP, Lai JS (2015). Patterns of genomic changes with crop domestication and breeding. Curr Opin Plant Biol.

[47] Reif JC, Zhang P, Dreisigacker S, Warburton ML, van Ginkel M, Hoisington D, Bohn M, Melchinger AE (2005). Wheat genetic diversity trends during domestication and breeding. Theor Appl Genet.

[48] Quarrie SA, Steed A, Calestani C, Semikhodskii A, Lebreton C, Chinoy C, Steele N, Pljevljakusic D, Waterman E, Weyen J, Schondelmaier J, Habash DZ, Farmer P, Saker L, Clarkson DT, Abugalieva A, Yessimbekova M, Turuspekov Y, Abugalieva S, Tuberosa R, Sanguineti MC, Hollington PA, Aragues R, Royo A, Dodig D (2005). A high-density genetic map of hexaploid wheat (*Triticum aestivum* L.) from the cross Chinese spring X SQ1 and its use to compare QTLs for grain yield across a range of environments. Theor Appl Genet.

[49] Kuchel H, Williams KJ, Langridge P, Eagles HA, Jefferies SP (2007). Genetic dissection of grain yield in breed wheat. I. QTL analysis. Theor Appl Genet.

[50] Zheng BS, Le GJ, Leflon M, Rong WY, Laperche A, Brancourt-Hulmel M (2010). Using probe genotypes to dissect QTL X environment interactions for grain yield components in winter wheat. Theor Appl Genet.

[51] Zhang JJ, Dell B, Biddulph B, Drake-Brockman F, Walker E, Khan N, Wong D, Hayden M, Appels R (2013). Wild-type alleles of *Rht-B1* and *Rht-D1* as independent determinants of thousand-grain weight and kernel number per spike in wheat. Mol Breed.

[52] Pearce S, Saville R, Vaughan SP, Chandler PM, Wilhelm EP, Sparks CA, Al-Kaff N, Korolev A, Boulton MI, Phillips AL, Hedden P, Nicholson P, Thomas SG (2011). Molecular characterization of *Rht-1* dwarfing genes in hexaploid wheat. Plant Physiol.

[53] Youssefian S, Kirby EJM, Gale MD (1992). Pleiotropic effects of the GA-insensitive *Rht* dwarfing genes in wheat. 2. Effects on leaf, stem, ear and floret growth. Field Crops Res.

[54] Ellis MH, Spielmeyer W, Gale KR, Rebetzke GJ, Richards RA (2002). “Perfect” markers for the *Rht-B1b* and *Rht-D1b* dwarfing genes in wheat. Theor Appl Genet.

[55] Börner A, Worland AJ, Plaschke J, Schumann E, Law CN (1993). Pleiotropic effects of genes for reduced height (*Rht*) and day-length insensitivity (*Ppd*) on yield and its components for wheat grown in middle Europe. Plant Breed.

[56] Addisu M, Snape JW, Simmonds JR, Gooding MJ (2010). Effects of reduced height (*Rht*) and photoperiod insensitivity (*Ppd*) alleles on yield of wheat in contrasting production systems. Euphytica.

[57] Worland AJ, Börner A, Korzun V, Li WM, Petrovic S, Sayers EJ (1998). The influence of photoperiod genes on the adaptability of European winter wheats. Euphytica.

[58] Foulkes MJ, Sylvester-Bradley R, Worland AJ, Snape JW (2004). Effects of a photoperiod-response gene *Ppd-D1* on yield potential and drought resistance in UK winter wheat. Euphytica.

[59] Liao XZ, Wang J, Zhou RH, Ren ZL, Jia JZ (2008). Mining favorable alleles of QTLs conferring 1000-grain weight from synthetic wheat. Acta Agronomic Sinica.

[60] Reeves TG, Rajaram S, van Ginkel M, Trethowan R, Braun HJ, Cassaday K (1999). New wheats for a secure, sustainable future. Mexico. D.f.: CIMMYT.

