# Supplementary figures and images for: Global status of 47 major wheat loci controlling yield, quality, adaptation and stress resistance selected over the last century
Source: BMC Plant Biol. 2019 Jan 3;19:5. doi: 10.1186/s12870-018-1612-y (PMC6318892; doi:10.1186/s12870-018-1612-y)

a

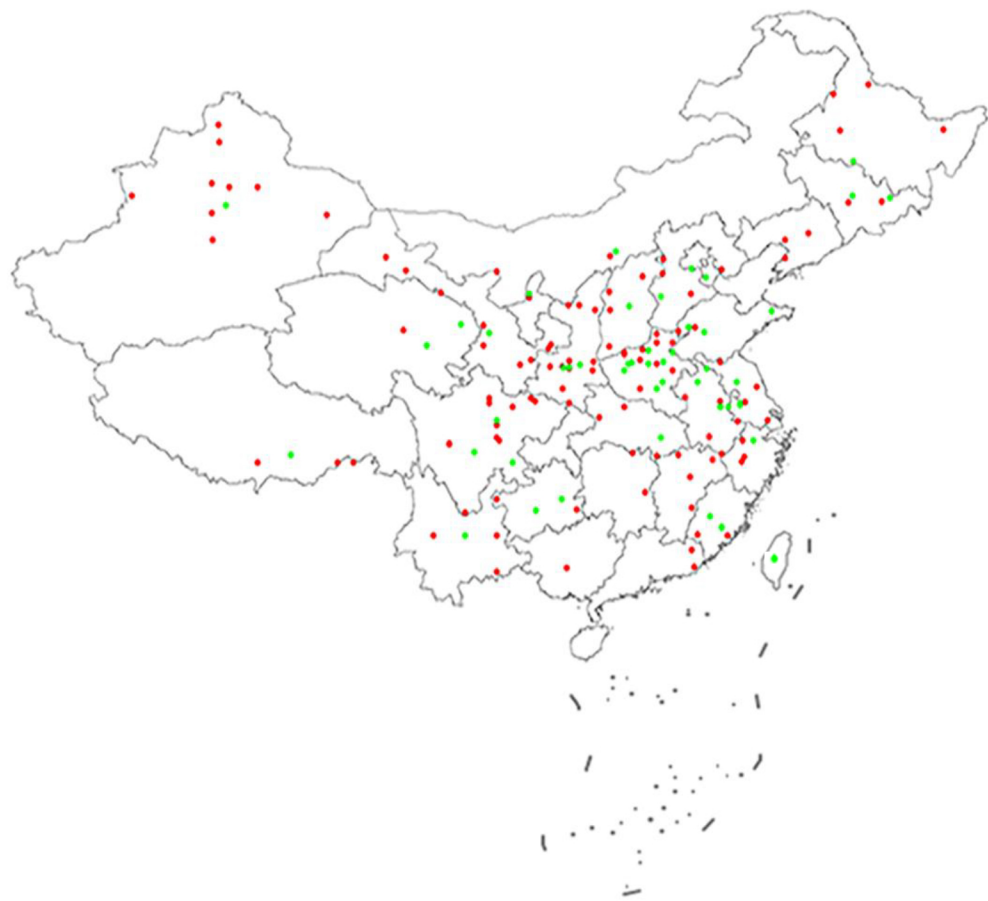

b

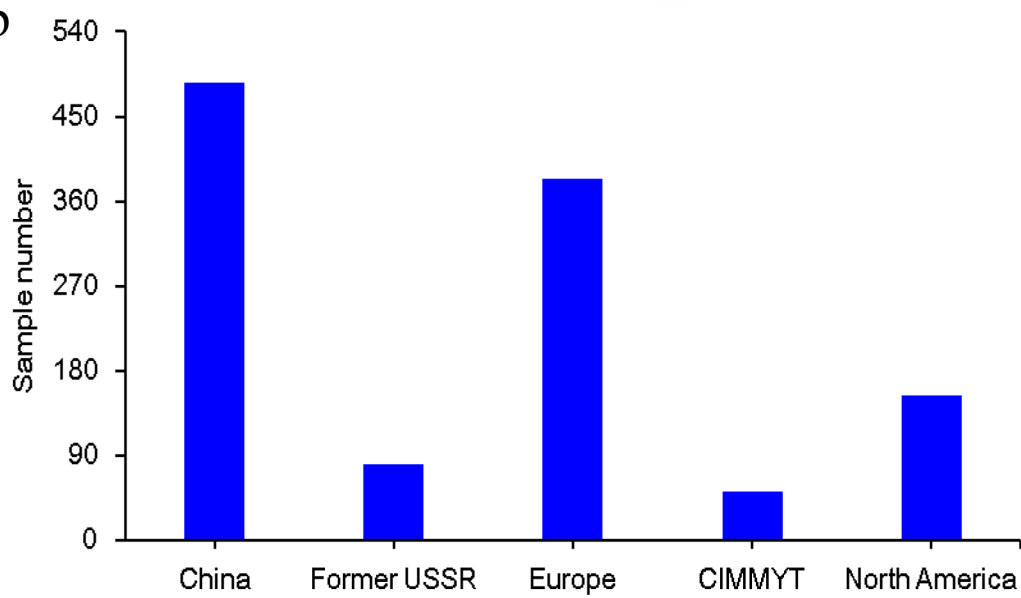

Supplement: Supplementary file 2 — Figure S1. Geographic origins of 1152 global wheat accessions used in the current study. a Geographic origins of Chinese wheat cultivars. Green shows modern cultivars; Red means landraces. The map of China is available at http://bzdt.nasg.gov.cn/. b The number of accessions in different subgroups. (PDF 2082 kb) [file 12870_2018_1612_MOESM2_ESM.pdf]

a

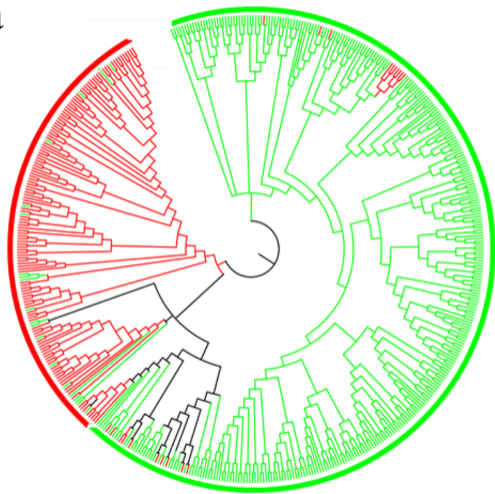

b

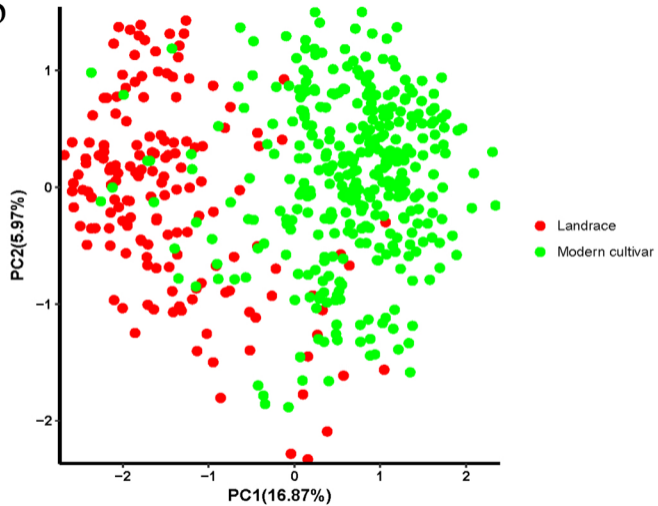

Supplement: Supplementary file 3 — Figure S2. Analysis of population structure in 480 Chinese wheat accessions based on 47 KASP markers. a Neighbor-joining tree of 323 modern Chinese cultivars and 157 Chinese landraces. Green and red lines represent modern Chinese cultivars and landraces, respectively. b PCA plots of 480 accessions based on the same markers. (PDF 2362 kb) [file 12870_2018_1612_MOESM3_ESM.pdf]

a

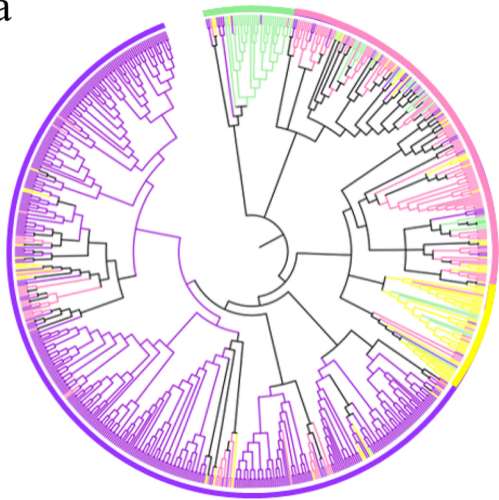

b

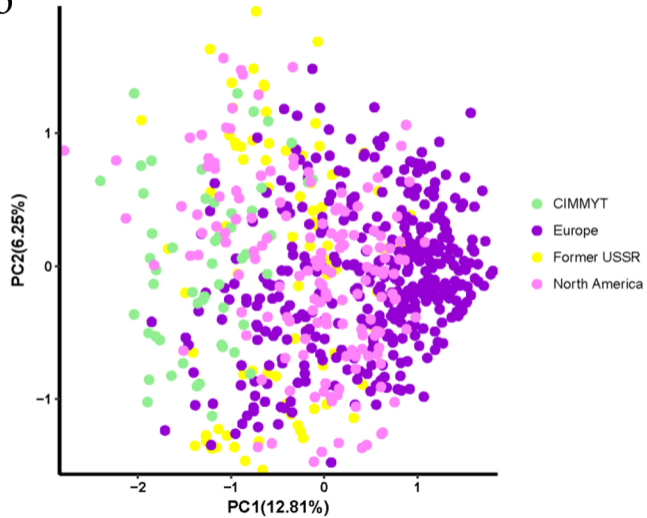

Supplement: Supplementary file 4 — Figure S3. Analysis of population structure in 672 introduced cultivars based on 47 KASP markers. a Neighbor-joining tree of 672 introduced accessions from four regions, including Europe, CIMMYT, the former Soviet Union (former USSR) and North America, marked in purple, green, yellow and pink, respectively. b PCA plots of 672 accessions based on the same markers. (PDF 2647 kb) [file 12870_2018_1612_MOESM4_ESM.pdf]

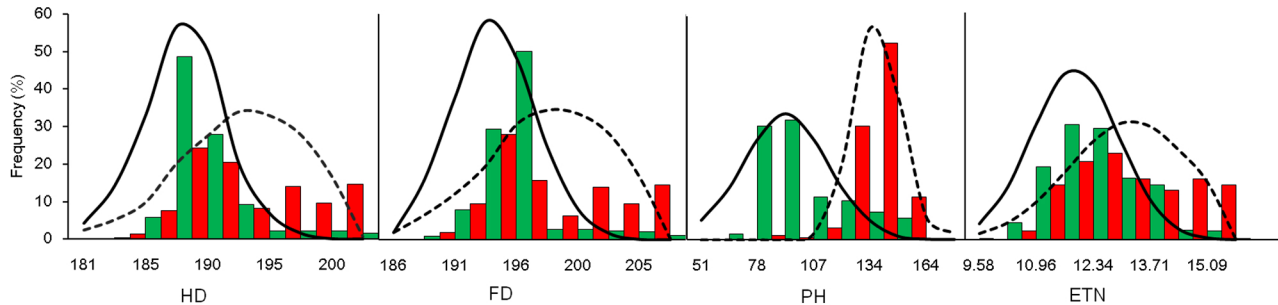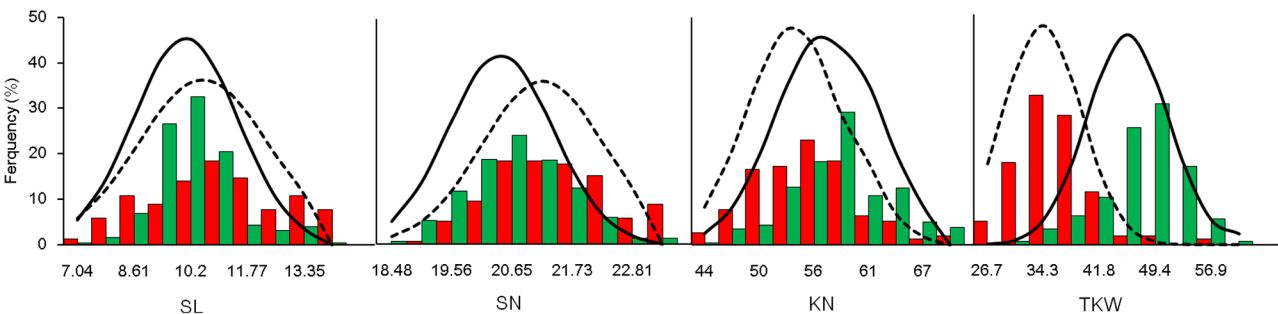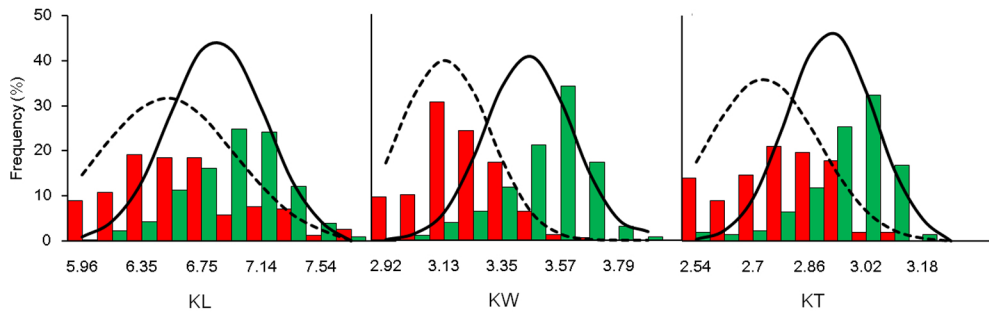

Supplement: Supplementary file 5 — Figure S4. Phenotypic variations of 11 agronomic traits in Chinese landraces (CL) and modern Chinese cultivars (MCC). Phenotypic values of CL and MCC are indicated by red histograms with a dashed black line and green histograms with a solid black line, respectively. HD: heading date (days); FD: flowering date (days); PH: plant height (cm); ETN: effective tiller number (number); SL: spike length (cm); SN: spikelet number per spike (number); KN: kernel number per spike (number); TKW: 1000-kernel weight (g); KL: kernel length (mm); KW: kernel width (mm); KT: kernel thickness (mm). (PDF 1928 kb) [file 12870_2018_1612_MOESM5_ESM.pdf]

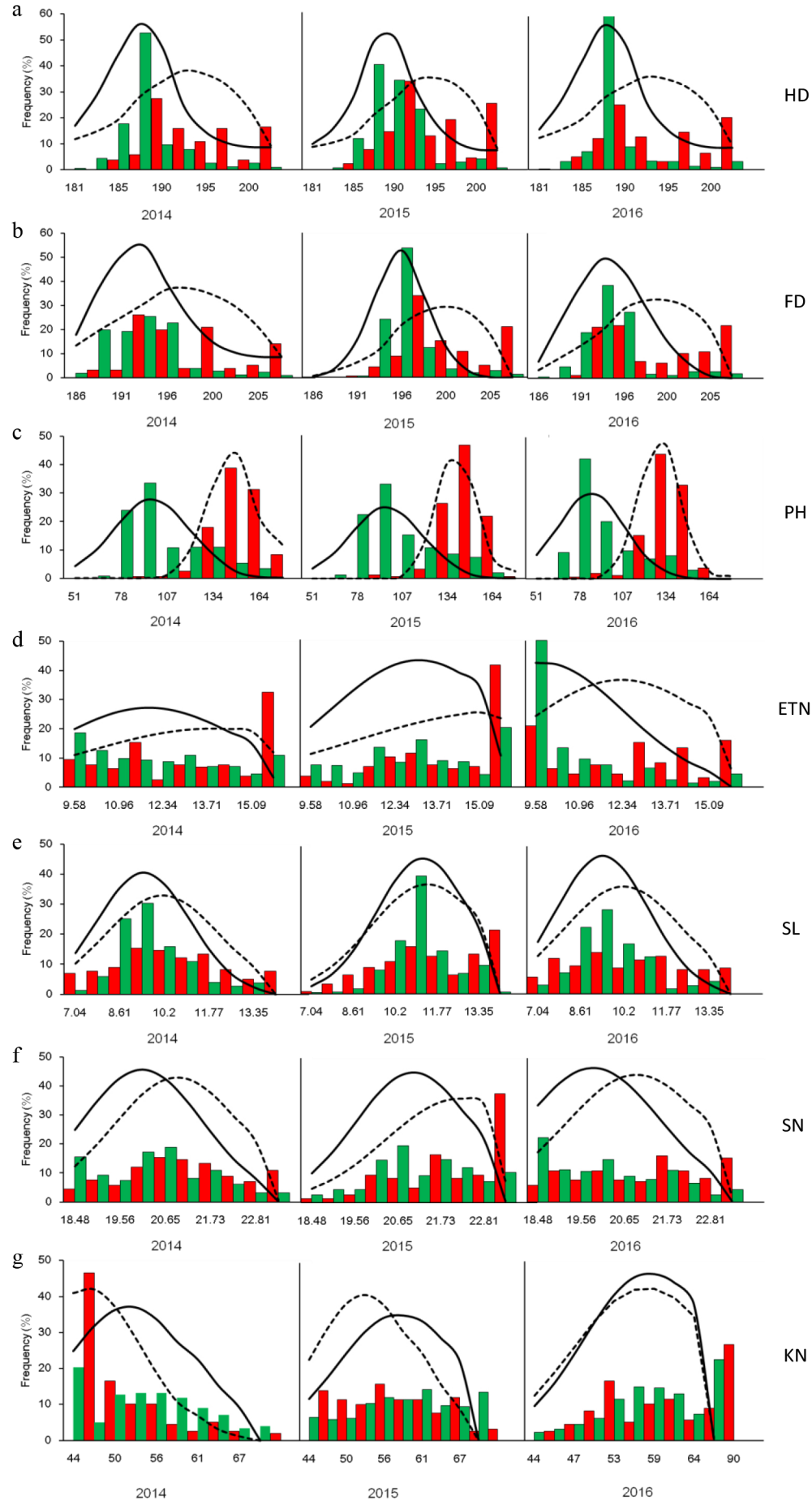

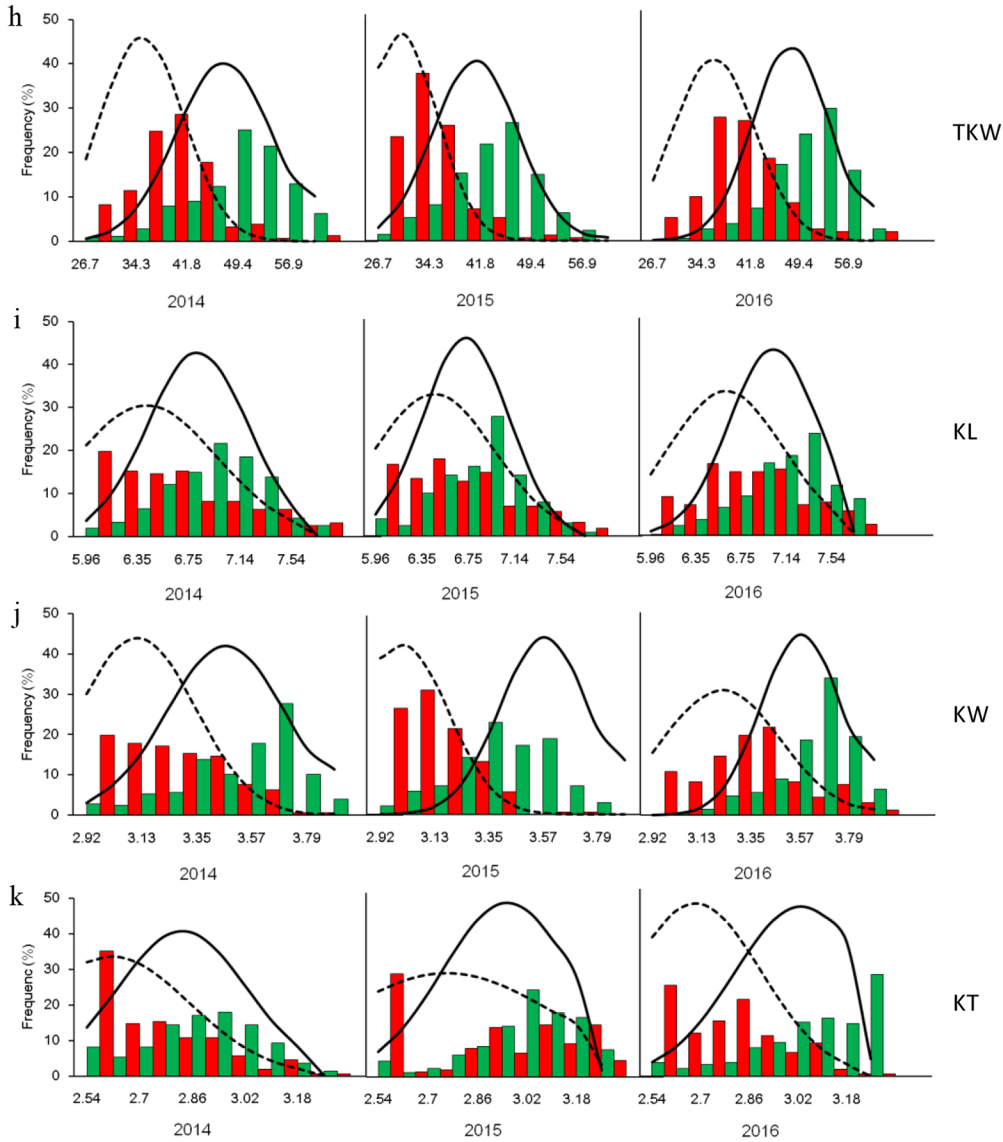

Supplement: Supplementary file 6 — Figure S5. Frequency distribution of phenotypic variations of 11 agronomic traits in Chinese landraces (CL) and modern Chinese cultivars (MCC) in three environments. Phenotypic values of CL and MCC are indicated by a red histogram with a dashed black line and a green histogram with a solid black line, respectively; 2014, 2015, and 2016 indicate the years in which all accessions were planted. a HD: heading date (days); b FD: flowering date (days); c PH: plant height (cm); d ETN: effective tiller number (number); e SL: spike length (cm); f SN: spikelet number per spike (number); g KN: kernel number per spike (number); h TKW: 1000-kernel weight (g); i KL: kernel length (mm); j KW: kernel width (mm); k KT: kernel thickness (mm). (PDF 5754 kb) [file 12870_2018_1612_MOESM6_ESM.pdf]

**a**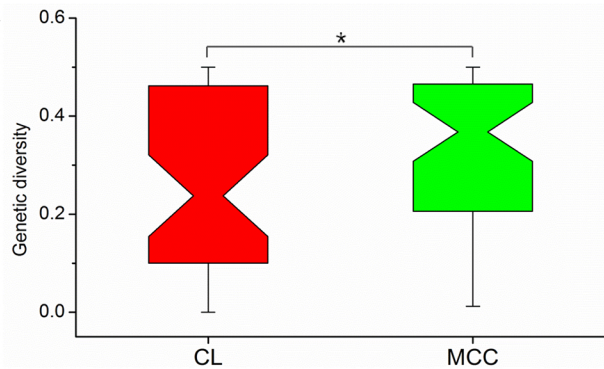**b**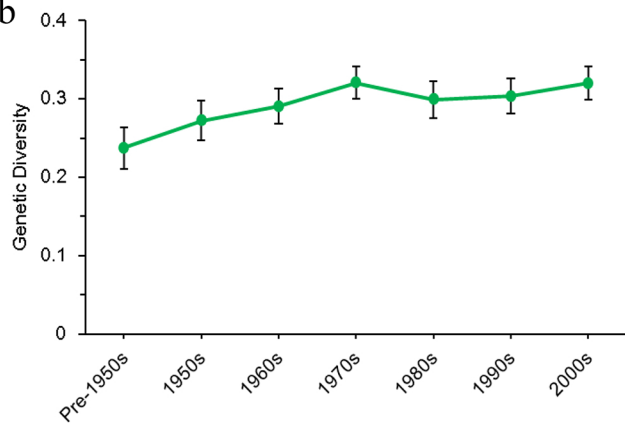**c**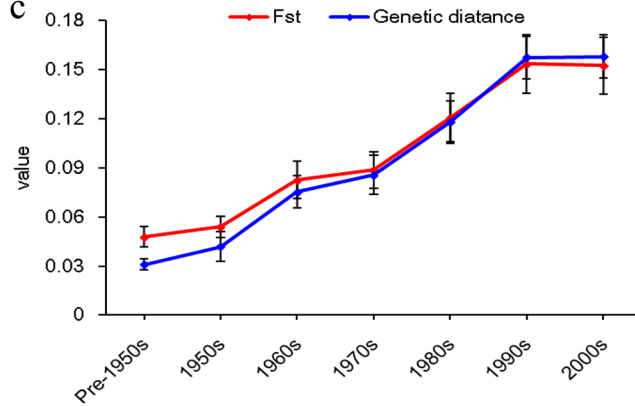

Supplement: Supplementary file 7 — Figure S6. Genetic diversity and population differentiation of Chinese landraces (CL) and modern Chinese cultivars (MCC). a Genetic diversity between CL and MCC. b Genetic diversity of the MCC during different decades. c Genetic differentiation and distance between CL and MCC during different decades. Fst and genetic distance are marked in red and blue, respectively. (PDF 2136 kb) [file 12870_2018_1612_MOESM7_ESM.pdf]

9

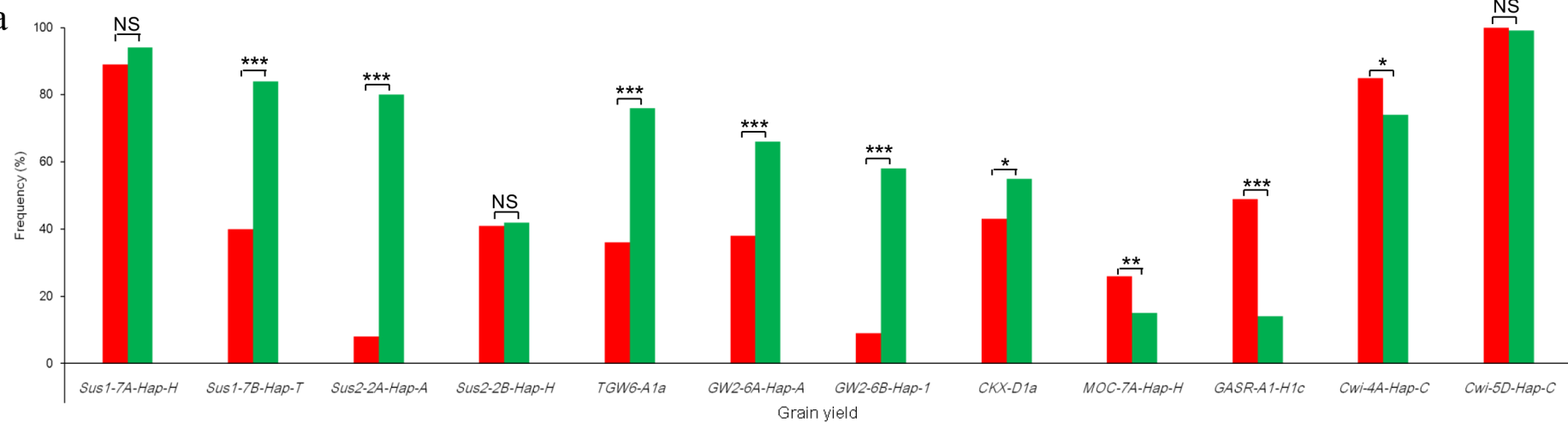

9

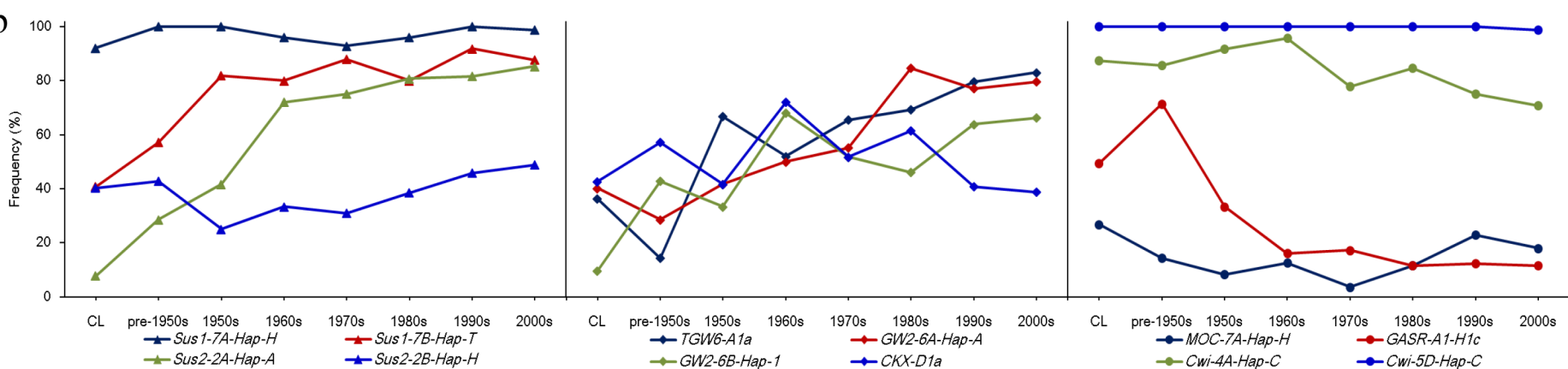

**c**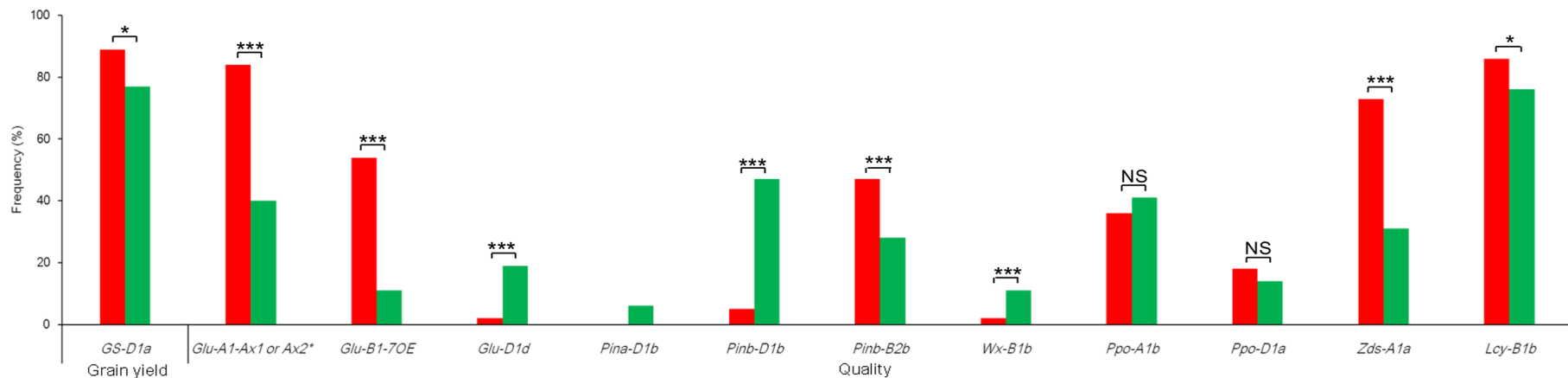**d**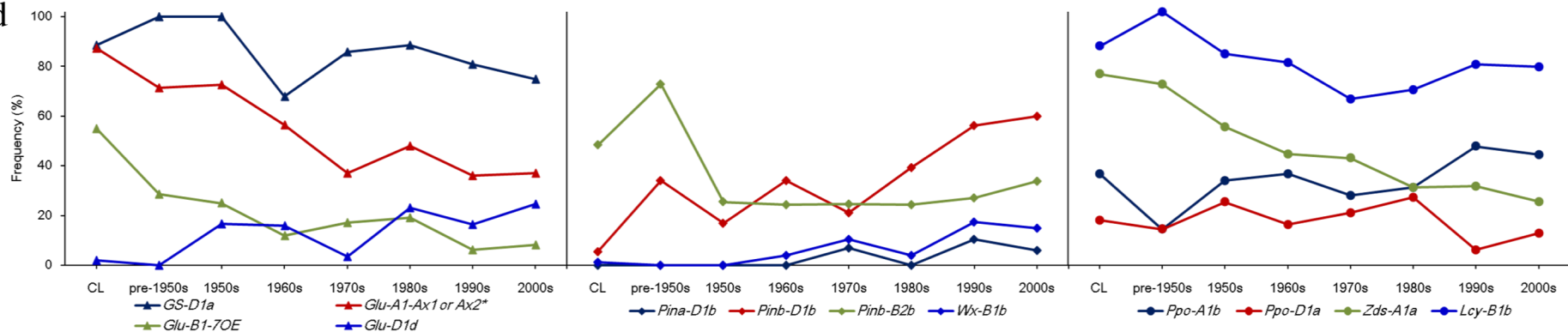

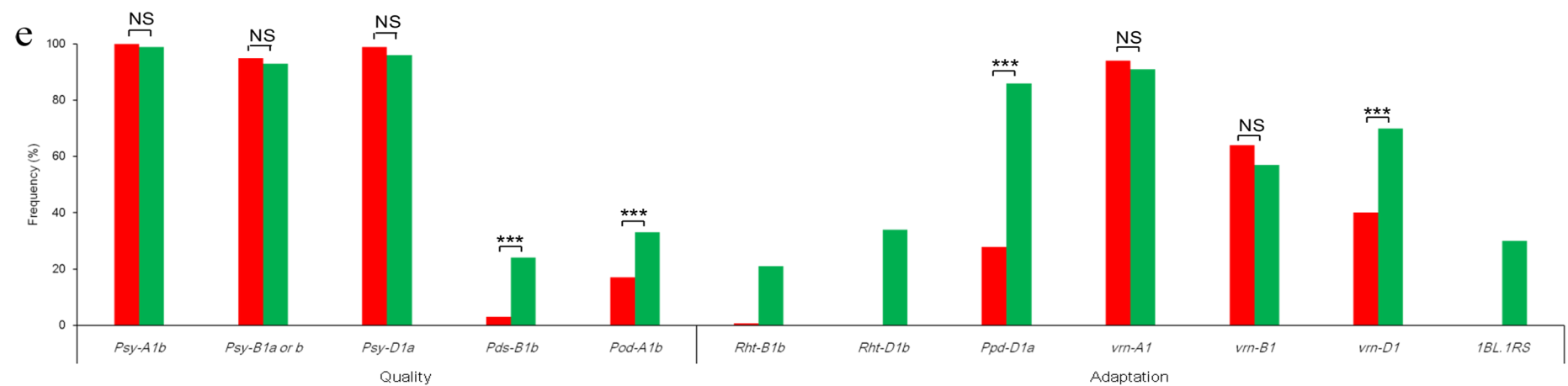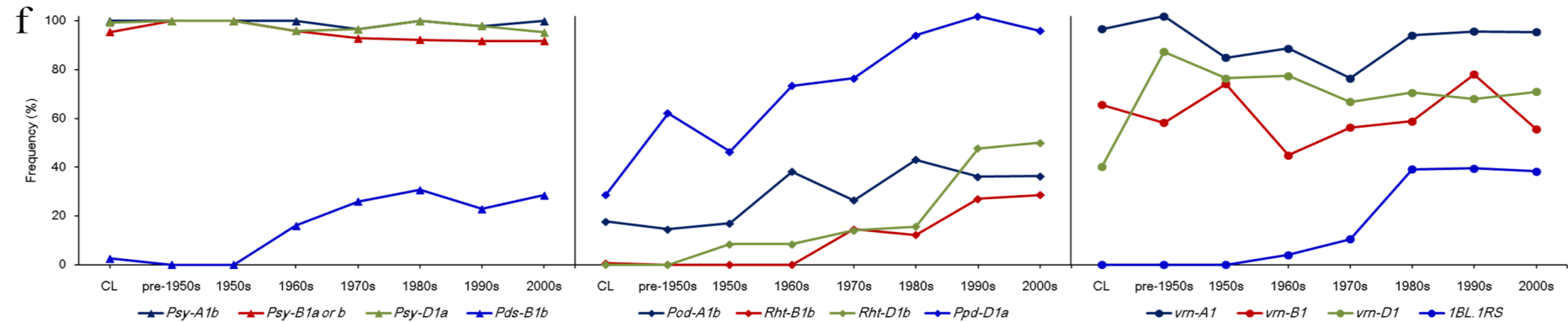

g

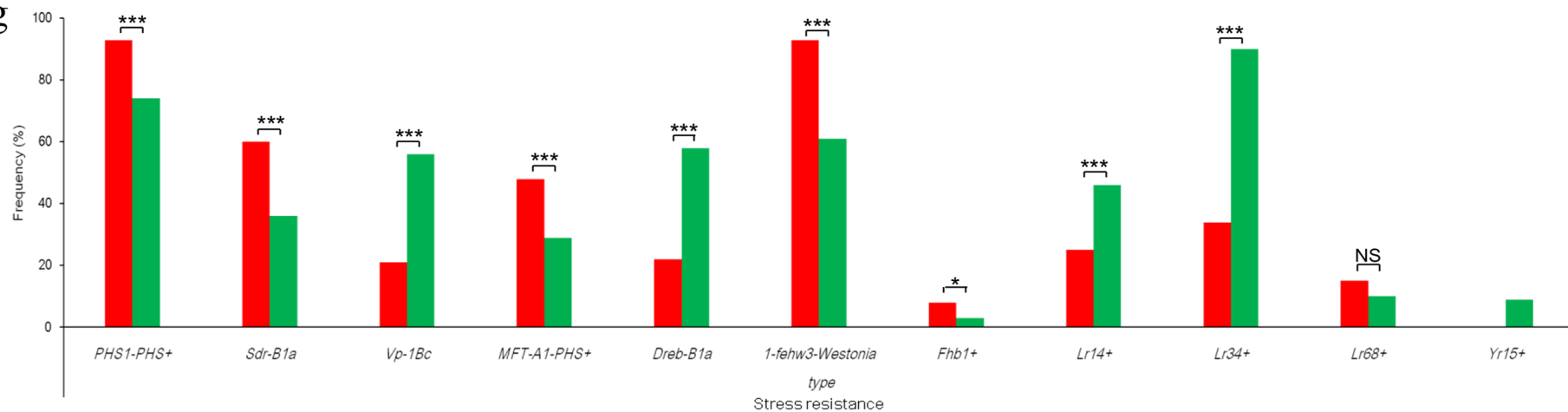

h

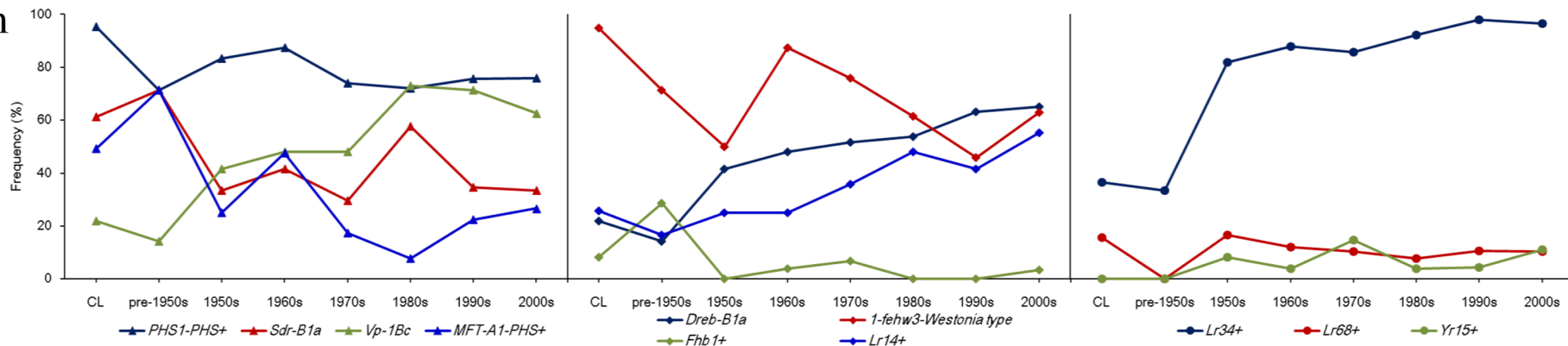

Supplement: Supplementary file 8 — Figure S7. Allele frequencies of 47 loci in Chinese landraces (CL) and modern Chinese cultivars (MCC). Red histogram shows CL, green histogram means MCC. a, c, e and g show allele frequencies in CL and MCC according to traits. b, d, f and h show changes in allele frequencies during different decades. *P<0.05; **P<0.01; ***P<0.001; NS, not significant. (PDF 8038 kb) [file 12870_2018_1612_MOESM8_ESM.pdf]

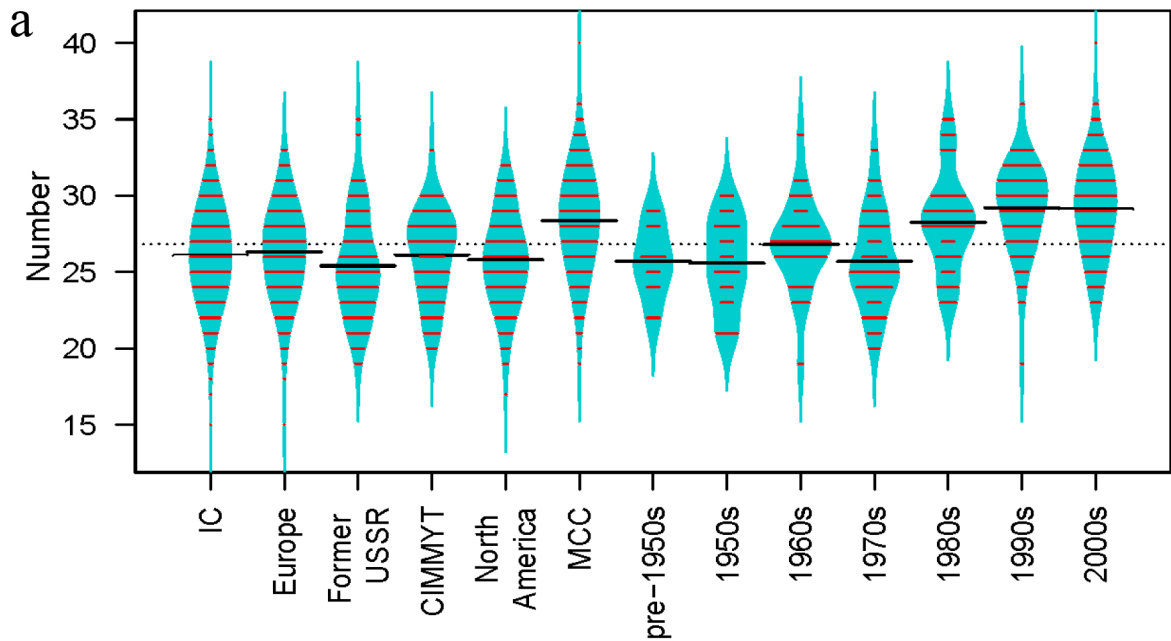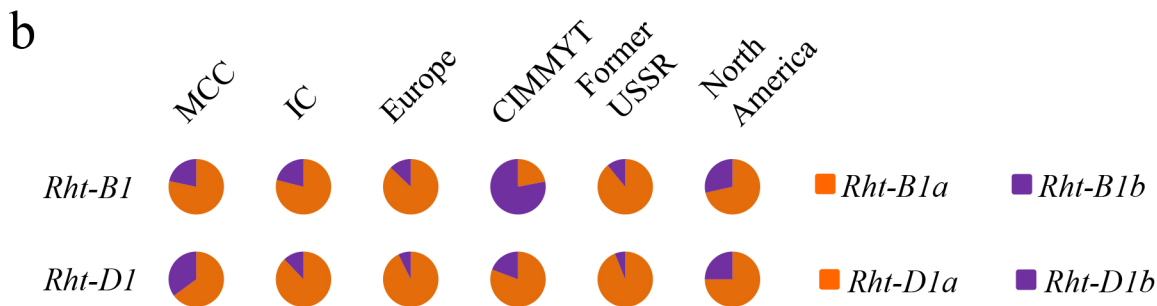

Supplement: Supplementary file 9 — Figure S8. Comparison of the numbers and frequencies of favorable alleles in global wheat accessions. a Bean plot for the distribution of favorable allele numbers in introduced cultivars (IC) from four regions and modern Chinese cultivars (MCC) during different decades. b Allele frequencies of Rht-B1 and Rht-D1 in different groups worldwide. (PDF 1151 kb) [file 12870_2018_1612_MOESM9_ESM.pdf]
